# Cell Lineage and Regional Identity of Cultured Spinal Cord Neural Stem Cells and Comparison to Brain-Derived Neural Stem Cells

**DOI:** 10.1371/journal.pone.0004213

**Published:** 2009-01-16

**Authors:** Theresa K. Kelly, Stanislav L. Karsten, Daniel H. Geschwind, Harley I. Kornblum

**Affiliations:** 1 The Semel Institute for Neuroscience and Behavior, David Geffen School of Medicine, University of California Los Angeles, Los Angeles, California, United States of America; 2 Mental Retardation Research Center, David Geffen School of Medicine, University of California Los Angeles, Los Angeles, California, United States of America; 3 Division of Neuroscience Research, Department of Neurology, Los Angeles Biomedical Research Institute at Harbor, University of California Los Angeles Medical Center, Torrance, California, United States of America; 4 Program in Neurogenetics and Department of Neurology, David Geffen School of Medicine, University of California Los Angeles, Los Angeles, California, United States of America; 5 Department of Human Genetics, David Geffen School of Medicine, University of California Los Angeles, Los Angeles, California, United States of America; 6 Departments of Molecular and Medical Pharmacology, Psychiatry, Pediatrics and Neurology, David Geffen School of Medicine, University of California Los Angeles, Los Angeles, California, United States of America; University of Maryland, United States of America

## Abstract

Neural stem cells (NSCs) can be isolated from different regions of the central nervous system. There has been controversy whether regional differences amongst stem and progenitor cells are cell intrinsic and whether these differences are maintained during expansion in culture. The identification of inherent regional differences has important implications for the use of these cells in neural repair. Here, we compared NSCs derived from the spinal cord and embryonic cortex. We found that while cultured cortical and spinal cord derived NSCs respond similarly to mitogens and are equally neuronogenic, they retain and maintain through multiple passages gene expression patterns indicative of the region from which they were isolated (e.g Emx2 and HoxD10). Further microarray analysis identified 229 genes that were differentially expressed between cortical and spinal cord derived neurospheres, including many Hox genes, Nuclear receptors, Irx3, Pace4, Lhx2, Emx2 and Ntrk2. NSCs in the cortex express LeX. However, in the embryonic spinal cord there are two lineally related populations of NSCs: one that expresses LeX and one that does not. The LeX negative population contains few markers of regional identity but is able to generate LeX expressing NSCs that express markers of regional identity. LeX positive cells do not give rise to LeX-negative NSCs. These results demonstrate that while both embryonic cortical and spinal cord NSCs have similar self-renewal properties and multipotency, they retain aspects of regional identity, even when passaged long-term in vitro. Furthermore, there is a population of a LeX negative NSC that is present in neurospheres derived from the embryonic spinal cord but not the cortex.

## Introduction

Neural stem cells (NSCs) self-renew and are multipotent, producing neurons, astrocytes and oligodendrocytes. As a consequence, NSC hold a great deal of promise for central nervous system repair [1]. A key question in the use of NSCs for neural repair is whether there are fundamental regional differences that dictate or constrain their capacity to differentiate into appropriate neuronal subtypes [2].

Early in development, the forebrain, midbrain, hindbrain and spinal cord delineate themselves from each other and continually become more specialized along the anterior-posterior and dorsoventral axes. The cellular basis of this regionalization is not well understood. One potential explanation is that the NSCs within a specific region are, or become, fundamentally distinct. Alternatively, regional differences within the CNS could be due to specialization at the stage of committed progenitors or differentiated cells. Prior studies have demonstrated at least some regional differences among NSC populations isolated from different CNS areas suggesting that there are multiple types of NSCs throughout the CNS [3], [4], [5]. These regional differences occur on many levels including proliferation [6], gene expression [3], [7], [8], the ability or likelihood of generating specific cell types [9], [10] and migration patterns [11]. If regional specialization takes place at the level of the stem cell, then NSCs isolated from a particular region will have intrinsic spatial information specific to that area which may limit their utility in neural repair to replace cells of that particular region. Indeed, heterotopic transplantation studies have demonstrated that some NSCs retain gene expression and/or differentiation ability of the region from which they were isolated suggesting intrinsic regional identity [12], [13]. However, there is also evidence that NSCs lose or gain abilities when isolated from their endogenous environment. For example, there is a loss of dorsoventral identity in cultured NSCs [11], [14], [15]. If this reprogramming and subsequent loss of dorsoventral identity occurs in vitro, it is possible that anterior-posterior identity information may also be lost when NSCs are cultured in vitro.

The goal of this study was to determine whether spinal cord and cortical derived NSCs have distinct intrinsic properties that suggest that they are regionally specified, and to determine whether such regional differences are maintained in vitro. We found that cortical and spinal cord derived NSCs have similar proliferative abilities in vitro. However, regional gene expression patterns are maintained in vitro, demonstrating that some aspects of regionally identity are maintained in vitro. Furthermore, while all NSCs derived from the embryonic cortex express LeX, there is a unique population of cells in the embryonic spinal cord that does not express LeX but fulfills the criteria of being a neural stem cell. This LeX negative NSC contains few of the identified markers of regional identity, but is able to generate LeX positive, regionalized NSCs, demonstrating that there is a lineal relationship between the two populations. These data demonstrate fundamental differences between embryonic spinal cord and brain derived- NSC and provide evidence for a previously unrecognized spinal cord NSC lineage.

## Results

### Cortical and spinal cord derived neurospheres share neural stem cell properties

**Figure 1:**
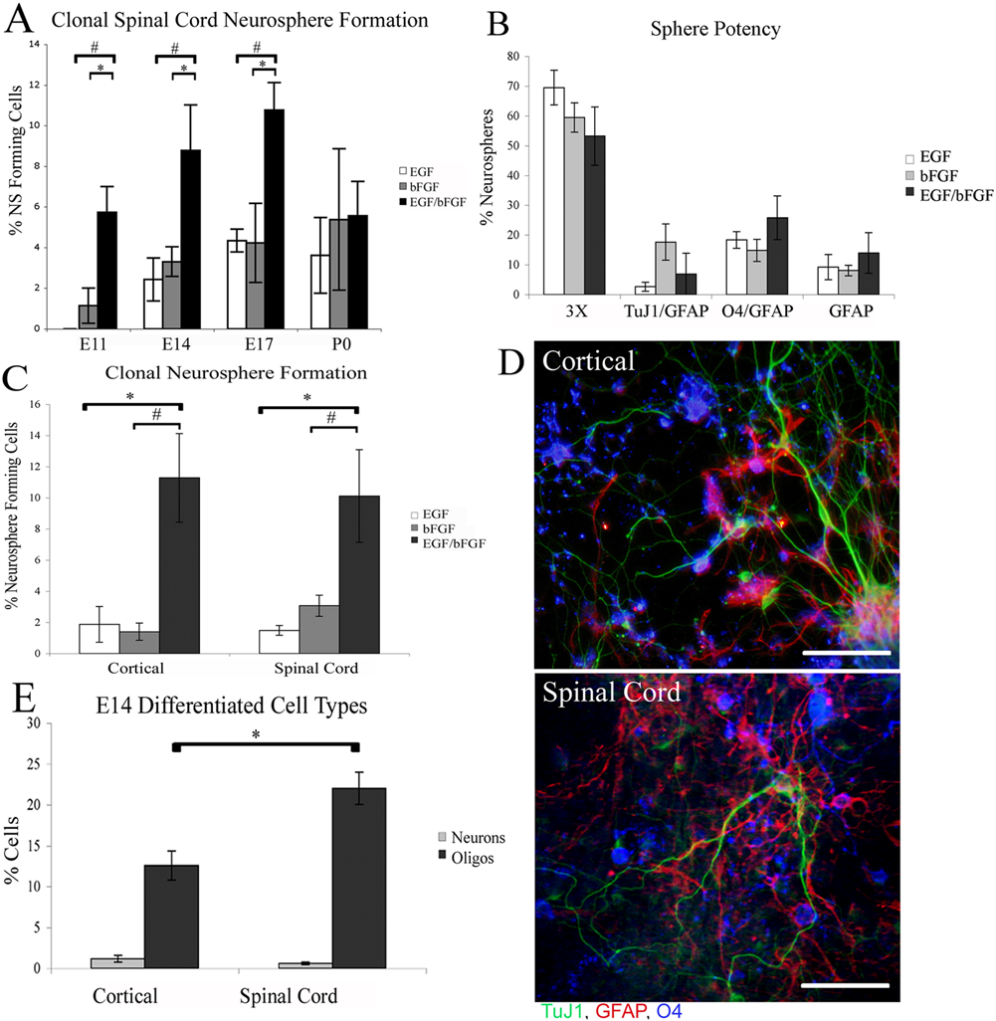
Spinal cord derived NSCs respond to mitogens in a similar fashion to cortical derived NSCs but produce more oligodendrocytes. (A) Secondary spinal cord clonal neurosphere formation from embryonic day 11, 14, 17 and post-natal day 0, in the presence of EGF or bFGF alone and in EGF and bFGF combined. (B) The percentage of E14 clonal secondary spinal cord derived neurospheres that contain cells that express markers of neurons, astrocytes and/or oligodendrocytes; (3×) indicates neurospheres containing all 3 cell types. (C) E14 secondary clonal neurosphere formation from cortical and spinal cord derived neurospheres in differing mitogen conditions. (D) Differentiated E14 clonal secondary cortical and spinal cord derived neurospheres express markers of neurons (TuJ1- Green), oligodendrocytes (O4- Blue) and astrocytes (GFAP- red). (E) Percentage of cells expressing Tuj1 (neurons) or O4 (oligodendrocytes) present in secondary embryonic day 14 differentiated cortical and spinal cord derived neurospheres. Bars are mean±SEM of at least 3 independent experiments. * P<0.05, # P<0.01, Anova followed by post hoc t-test. Scale bar in D: 110 µm.

**Figure 2:**
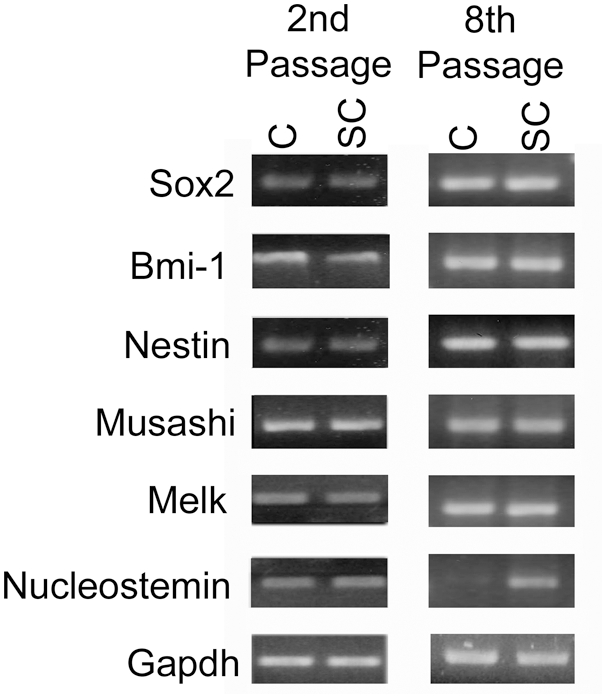
Stem cell associated genes are expressed in cortical and spinal cord derived neurospheres and maintained for multiple passages: RT-PCR on E14 cortical and spinal cord derived neurospheres that had been cultured in EGF and bFGF for 2 and 8 passages. Abbreviations: Cortical (C) Spinal Cord (SC).

One characteristic of NSCs is the ability to self-renew. The ability to passage neurospheres clonally is an indicator of self-renewal. Cortical and spinal cord derived neural stem cells displayed similar growth factor responsiveness throughout embryonic development (Figure 1). The majority of clonal neurospheres generated from the E14 spinal cord were tripotent (producing neurons, astrocytes and oligodendrocytes), and equally neuronogenic, but more oligogenic relative to cortical derived NSCs (Figure 1). We did not detect any specific mature neuronal subtypes in our differentiated cortical or spinal cord cultures (data not shown), however this is likely a result of differentiating progenitors in minimal media for limited time. Next, we asked whether spinal cord progenitors expressed similar sets of stem cell-associated genes as cortical derived progenitors. RT-PCR analysis of cortical and spinal cord derived neurospheres demonstrated that both expressed Sox2 (SRY-related HMG-box gene 2) [16], Bmi-1 (B-cell-specific Moloney murine leukemia virus integration site 1) [17], Nestin [18] Musashi-1, [19] and Melk (Maternal embryonic leucine zipper kinase) [20] to a similar degree, whether measured at early or late passages (Figure 2). Nucleostemin [21] expression was roughly equivalent in cortical and spinal cord neurosphere cultures at early passages and became enriched in spinal cord derived neurospheres at later passages. The majority of cells within cortical and spinal cord secondary neurospheres expressed LeX and/or nestin (Figure S1). Thus, embryonic spinal cord and cortical neurospheres express similar sets of genes previously shown to underlie fundamental aspects of neural stem cell biology, implying, as might be expected, a common regulation of both cortical and spinal cord NSCs.

### NSCs maintain anterior-posterior regional identity in vitro

**Figure 3:**
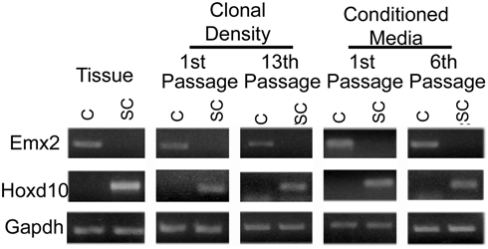
Regional gene expression is maintained in vitro and is cell intrinsic. RT-PCR of Emx2 and Hoxd10 expression in E14 cortical and spinal cord tissue and neurospheres at first and 13^th^ passage (clonal density). Emx2 and Hoxd10 expression in E14 cortical and spinal cord neurospheres that had been cultured in media that was conditioned by neurospheres from the opposing region and passaged 1 and 6 times (conditioned media).

In vivo, Emx2, (empty spiracles 2), expression is restricted to the telencephalon [22], while Hoxd10, a homeobox gene, is expressed in the caudal neural tube [23], [24]. We used these genes as markers of regional identity and found that their differential expression was maintained in vitro after multiple passages at clonal density (Figure 3). Although the degree to which NSC could interact is limited when cultured at low density, it is possible that they secrete soluble factors to maintain their regional identities. In order to test this possibility, we used cortical or spinal cord neurosphere-conditioned medium. We found that cortical-derived neurospheres continued to express Emx2 when cultured in media conditioned by spinal cord neurospheres and spinal cord derived neurospheres continued to express Hoxd10 when cultured in media conditioned by cortical neurospheres (Figure 3). These results further demonstrate that regional identity of NSCs is established by embryonic day 14 and is an intrinsic property of the NSC. To determine whether spinal cord derived neurospheres were biased along the dorsoventral axis, we examined the expression of genes indicative different dorsoventral domains. We detected the expression of genes indicative of multiple dorsoventral domains demonstrating that spinal cord derived neurospheres were not dorsally or ventrally biased (Figure S2).

### Significant gene expression differences exist among neural stem cells from different regions of the CNS

In order to further examine differences between spinal cord and cortical- derived NSC, we compared neurosphere gene expression by microarray analysis. Using the stringent criteria outlined in Methods, we identified 229 genes that were differentially expressed between cortical and spinal cord derived neurospheres. One hundred fifty genes were enriched in spinal cord derived neurospheres while 79 genes were enriched in cortical derived neurospheres (Supplemental Table 2). A subset of differentially expressed genes and their corresponding GO biological process category is shown in Table 1. Amongst the most enriched genes, there were a striking number of homeodomain genes, with a greater number enriched in spinal cord- than in cortical- derived neurospheres.

**Table 1:** A subset of differentially expressed genes based on the GO Biological Process Category based on DAVID/EASE analysis.

GO Biological Process Gene Category	Spinal Cord Enriched	Cortical Enriched
apoptosis	Agtr2; Card4; Cxcr4; Dcc; Gadd45G; Pax3; Pde1B; Sgk	Cdkn1a; Dapk2; Pak1; Pmaip1; Tnfrsf19
cell adhesion	Chl1; Col16a1; Itga8; Itga9; Lama4; Loxl2; Lrrn1; Nrxn3; Pcdh15; Pcdh17; Pcdh9; Pcdha6; Pcdha9; Pgm5; Pkp2; Reln; Sema5a; Slit2; Tnc	Cd44; Cdh4; Clstn2; Col18a1; Col4a1; Efs; Mfge8; Nptx2; Ntn4
cell cycle	Cdk6; Dcc; Igf2; Lck; Tep1	Bcar3; Bcat1; Ccng1; Cdkn1A; Cdkn2B; Junb; Lyn; Mgmt; Smc5l1
cell motility	Adra2a; Atp1a1; Nrxn3; Prkg1; Slit2	Dmd; Myh7; Nebl
cell organization and biogenesis	Adra2a; Hmga2; Igf2; Kif5a; Prkg1	Myh7; Nebl
cell proliferation	Adra2a; Cdk6; Dcc; ENPEP; Hoxc10; Igf2; Lck; Odz2; Tep1	Bcar3; Bcat1; Ccng1; Cdkn1A; Cdkn2B; Col18A1; Egfr; EgR4; Junb; Lyn; MGMT; Pmp22; Rarres1; Smc5l1; Tgfbr2
cell surface receptor linked signal transduction	Abcg2; Adra2a; Agtr2; Calca; Ccrl1; Cxcr4; Dll3; Epha7; Gabra4; Gabrb1; Gabrg1; Gap43; Gnb4; Gpr45; Gpr49; Grb10; Grid2; Igf2; Itga8; Itga9; Lphn3; Oprl1; Pdgfrl; Ptprd; Trhr; Wnt3	Bmp7; Egfr; Gpr37; Grin2c; Ntrk2; Rgs7; Tgfbr2
cell-cell signaling	Bmp3; Calca; Cpne6; Enpep; Fgf13; Gabra4; Gabrb1; Gabrg1; Grb10; Grid2; Kif5a; Pace4; Sema5a; Wnt3	Efnb2; Gchfr; Gria2; Grin2c; Pmp22
G-protein coupled receptor protein signaling pathway	Abcg2; Adra2a; Agtr2; Calca; Ccrl1; Cxcr4; Gabra4; Gabrb1; Gabrg1; Gap43; Gnb4; Gpr45; Gpr49; Grb10; Grid2; Lphn3; Oprl1; Trhr	Gpr37; Grin2c; Rgs7
morphogenesis	Anxa2; Bmp3; Calca; Cpne6; Cxcr4; Dcc; Dcn; Dll3; Dpysl4; Fgf13; Gap43; Gbx2; Hey2; Hoxa4; Hoxa11; Hoxb5; Hoxc10; Igf2; Matn3; Msx1; Nkx6-1; Nrxn3; Olfm1; Pax3; Pnma2; Pvalb; Runx2; Sema5a; Six1; Slit2; Tuft1; Wnt3	Angpt2; Apba2; Bmp7; Col18a1; Dab1; Dcamkl1; Dmd; Dscr1l1; Efnb2; Epas1; Eya1; Gypc; Lhx2; Myh7; Nr2e1; Nr4a2; Ntrk2; Otx2; Pmp22
neurogenesis	Cpne6; Cxcr4; Dcc; Dll3; Dpysl4; Fgf13; Gap43; Gbx2; Hey2; Nrxn3; Olfm1; Pax3; Pnma2; Sema5a; Slit2	Apba2; Dab1; Dcamkl1; Dscr1l1; Efnb2; Lhx2; Nr2e1; Nr4a2; Ntrk2; Otx2; Pmp22
pattern specification	Hoxa1; Hoxa2; Hoxa3; Hoxa5; Hoxa9; Hoxa11; Hoxb3; Hoxb5; Hoxb6; Hoxb7; Hoxc8; Hoxc9; Hoxc10; Hoxd4	Bmp7; Zic3
regulation of cell growth	Gap43	Igfbp3

All genes listed reached criteria for differential expression: >2 fold change in expression in each hybridization with p<0.01.

Consistent with our RT-PCR data, we did not find significant differences amongst a number of genes that have previously been associated with NSC or self-renewal capacity. These genes include Nestin, Sox2, Bmi1, Musashi, Melk, and Nucleostemin [16], [17], [19], [20], [21], [25], [26]. An exception to this was Abcg2, which was enriched in spinal cord derived neurospheres relative to cortical derived neurospheres.

In order to more formally analyze the differences in gene expression between the different neurosphere populations, we examined their gene ontology using DAVID/EASE (https://apps1.niaid.nih.gov/david) [27]. Several functional categories were identified including development, regulation of cellular process, morphogenesis, metabolism, transcription and neurogenesis. Gene Ontology analysis demonstrated that most significantly enriched functional categories (EASE score<10^−8^) are related to development (20%), pattern specification (5%), morphogenesis (11%), CNS development (7%), neurogenesis (7%), and neuron differentiation (5%; Figure S3). Thirty-seven genes belonged to the pattern specification group, supporting our conclusion that spinal cord and cortical neurospheres retain their regional identity in vitro, Eighty four percent of these genes are transcription factors. The largest group (n = 21) belong to the Hox-family of homeobox transcriptional regulators and many are known to be involved in spinal cord patterning [28]. All of the identified Hox genes are highly enriched (from 4 to 45-fold) in the spinal cord derived neurospheres, again consistent with the hypothesis that key regulatory factors are preserved *in vitro*. Other transcriptional factors involved in brain development or neural stem cell differentiation included Nr2e1 (Tlx), Emx2 (empty spiracles homolog 2), Foxg1 (forkhead box g1), Otx2 (orthodenticle homolog 2), Pax3 (paired box gene 3), Dll3 (Delta-like 3), Gbx1 (gastrulation brain homeobox 1), Lhx2 (lim homeobox protein 2), Pbx3 (pre b-cell leukemia transcription factor 3), Tbx18 (t-box18) and Zic3 (zinc finger protein of the cerebellum 3). Interestingly, Nr2e1 was 50 fold enriched in cortical derived neurospheres and has previously been shown to regulate embryonic and adult cortical neurogenesis [29], [30]. Emx2 functions in combinations with Otx2 to specify cell fates in the developing telencephalon [23], [31]. Emx2 and Otx2 were 10 and 5 fold enriched in cortical-derived neurospheres, respectively (Supplemental Table 2). Foxg1 was 10-fold enriched in cortical-derived neurospheres consistent with its role in the development of the telencephalon [32].

In order to confirm and extend the microarray data, we used quantitative RT-PCR. We were able to confirm the differential expression of 15/15 genes chosen because they represented a broad range of expression differences on the microarray (Table 2). We next determined whether differential expression of genes identified by microarray was maintained over multiple passages. Thirteen out of fifteen (86.67%) genes examined remained differentially expressed over 13 passages (Table 2). These data further demonstrate that cortical and spinal cord derived neural stem and progenitors retain at least some aspects of their regional identity in vitro.

**Table 2:** Confirmation of differential gene expression based on region by qRT-PCR.

		Ratio from Microarray	E14 secondary spinal cord NS relative to E14 secondary cortical NS	E14 13th passage spinal cord NS relative to E14 13th passage cortical NS	E14 secondary spinal cord NS relative to E11 secondary cortical NS	E14 secondary spinal cord NS relative to E17 secondary cortical NS
Cortical Enriched Genes	**Lhx2**	**0.149**	**0.108**	**0.485**	**0.076**	**0.053**
	**Nr2e1**	**0.019**	**0.001**	**0.038**	**0.003**	**0.002**
	**Emx2**	**0.129**	**0.012**	**0.166**	**0.005**	**0.898**
	**Arx**	**0.28**	**0.524**	**0.527**	**0.499**	**0.223**
	Egf-R	0.292	0.006	0.093	0.002	1.072
	**Ntrk2**	**0.416**	**0.543**	**0.484**	**0.508**	**0.278**
	Ccng1	0.445	0.817	0.442	0.402	1.159
Spinal Cord Enriched Genes	**Hoxc10**	**5.221**	**634.448**	**678.059**	**367.427**	**470.030**
	Pace4	5.212	2.480	0.708	12.569	1.591
	**Irx3**	**3.21**	**21.470**	**4.846**	**22.765**	**8.692**
	Anxa2	2.596	3.447	0.506	1.217	1.794
	**Abcg2**	**2.45**	**5.202**	**2.100**	**2.395**	**4.658**
	**Cav2**	**2.409**	**2.549**	**3.174**	**4.329**	**1.537**
	Fut9	2.225	2.908	3.401	1.295	0.174
	**Mro**	**2.002**	**1.905**	**1.161**	**2.487**	**1.078**

RNA was extracted from neurospheres derived from E14 cortex and spinal cord and E11 and E17 cortex. The following comparisons were made: Secondary E14 cortical and spinal cord derived neurospheres, E14 cortical and spinal cord neurospheres after 13 passages, E11 cortical derived and E14 spinal cord derived neurospheres, E17 cortical and E14 spinal cord derived neurospheres. Results represent the delta delta critical threshold of cortical derived neurospheres compared to spinal cord derived neurospheres. Genes that maintain their differential expression between cortical and spinal cord neurospheres through all conditions are in bold. The first seven genes were identified by microarray as enriched in cortical derived neurospheres. The bottom eight genes were identified by microarray as enriched in spinal cord derived neurospheres. Relative expression>1 indicates greater expression in spinal cord derived neurospheres.

The central nervous system does not develop at the same time for all regions. Within the rodent spinal cord, there is a rostrocaudal gradient, with rostral portions developing earlier than posterior segments [33], [34]. The degree to which this developmental gradient holds for the entire neuraxis is not clear, however. In order to determine whether gene expression differences between cortical and spinal cord neurosphere were due to different developmental timing, we compared gene expression in E14 spinal cord derived neurospheres to cortical- derived neurospheres from E11 and E17 (Table 2). All of the genes tested maintained their differential expression when E11 cortical derived neurospheres were compared to E14 spinal cord derived neurospheres, while twelve of fifteen genes (80%) maintained their differential expression when E17 cortical derived neurospheres were compared to E14 spinal cord derived neurospheres (Table 2). We found that ten of the fifteen (67%) genes tested were also differentially expressed when primary tissue was compared, suggesting the identified genes are not simply an artifact of cell culture (Supplementary Table 3). Genes that maintained their differential expression across all time points, likely reflect region-specific differences between cortical and spinal cord- derived progenitors while genes whose differential expression is not maintained may reflect a biological process that is going on at a particular time in development.

### There is a unique, LeX negative neural stem cell population present in the embryonic spinal cord

**Figure 4:**
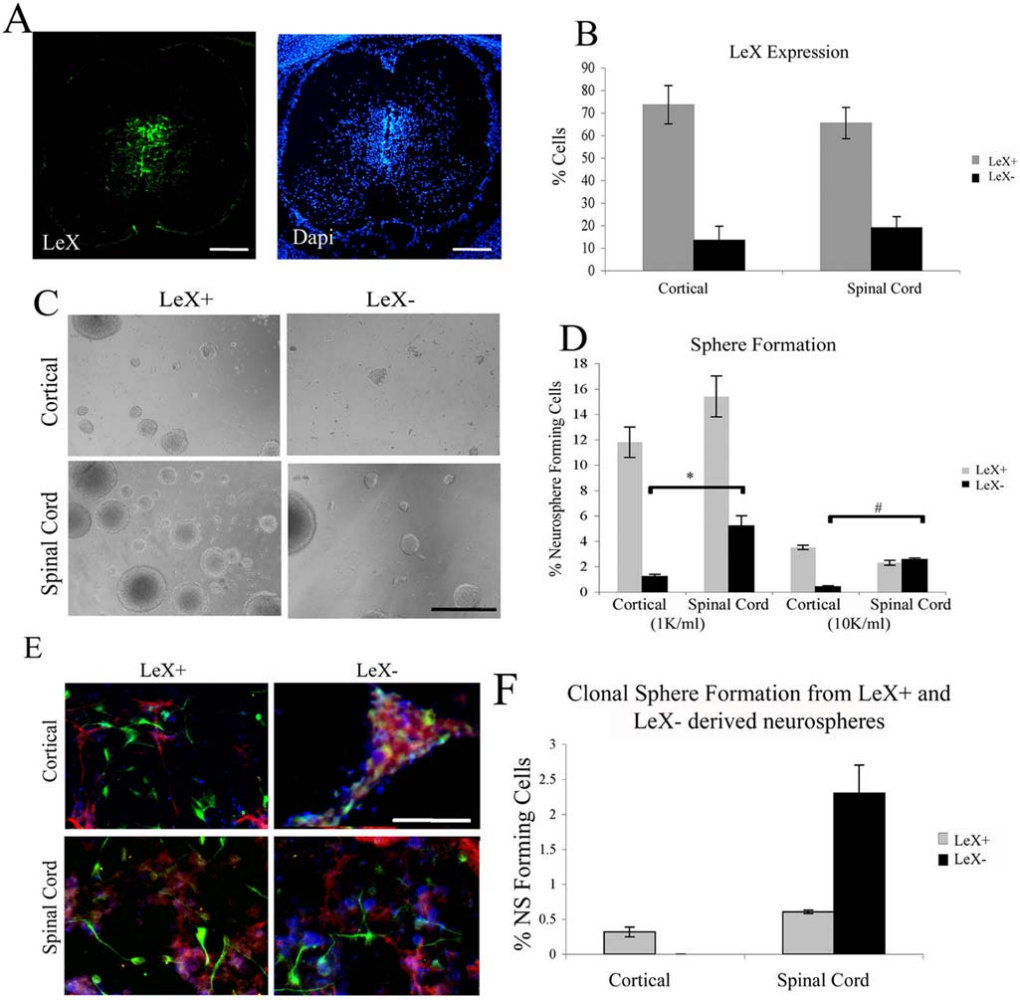
LeX negative cells from spinal cord, but not cortical derived neurospheres are neural stem cells. (A) LeX expression in a coronal section of the embryonic day 15 spinal cord; LeX (Green) Dapi (Blue); Dorsal (top) ventral (bottom). (B) Percentage of cells in secondary neurospheres derived from cortex and spinal cord that express LeX. (C) Photomicrographs of spheres generated from E14 cells following sorting for LeX expression (at second passage). LeX negative cells from cortical derived neurospheres tend to form clusters rather than round phase bright neurospheres. (D) Quantification of neurosphere formation from E14 cortical and spinal cord derived cells following sorting. Sorted cells were cultured at clonal density (1,000 cells/ml) and 10,000 cells/ml. (E) Immunocytochemistry of differentiated E14 clonal neurospheres generated by LeX expressing and non expressing cells from cortical and spinal cord derived neurospheres. Upon differentiation, clusters generated by LeX negative cells from cortical neurospheres lose cell integrity and do not generate morphologically distinct cell types. TuJ1 (Green), O4 (Blue), GFAP (Red). (F) Ability of cells within a sphere generated by LeX+ and LeX− cells to form a new neurosphere. Bars are mean±SEM of at least 3 independent experiments. * P<0.001, # P<0.0000005, Anova followed by post hoc t-test. Scale bar in A: 450 µm D: 200 µm and in E: 110 µm.

Lewis X (LeX, SSEA-1, CD15) is a carbohydrate moiety that is expressed by a subset of nestin positive progenitors [35], [36] as well as some differentiated cells [37], [38]. While LeX negative cells do not generally form neurospheres, neurospheres are formed from a subset of LeX positive cells when they are derived from adult subventricular zone (SVZ), embryonic forebrain germinal zone [39], or cultured SVZ-derived GFAP-expressing progenitors [40]. LeX is expressed in the embryonic spinal cord in the area surrounding the central canal, where spinal cord neural stem and progenitor cells are located ([38], [39] and Figure 4a). We examined neurosphere cultures for LeX and found it to be expressed by the majority of cells within neurospheres from different regions of the CNS and LeX expression did not differ based on region of isolation (73.59%±8.5 cortical and 65.44%±6.92 spinal cord) (Figure 3b).

As expected, we found that neurosphere forming capability resided almost exclusively within the LeX positive fraction in cultured cortical cells. However, surprisingly, when cultured at relatively high or low densities, both LeX positive and LeX negative spinal cord derived cells generated neurospheres (Figure 4c,d). The spheres derived from LeX negative spinal cord cells had the typical round, phase-bright appearance characteristic of neurospheres. On the other hand, LeX negative cells from the cortex did not form typical neurospheres, but rather clustered together and adhered to the flask, making it likely that the graph shown in Figure 4d is an overestimate of the number of true neurospheres formed from LeX negative cortical derived cells. At clonal density, the sphere forming capacity of spinal cord derived LeX positive cells was greater than that of LeX negative cells, while at higher density this capacity was the same. This could be due to multiple cells sticking together to form a single sphere at higher densities or the previously described mutual inhibition of LeX positive cells on the formation of neurospheres [35], [36].

Neurospheres from LeX negative spinal cord cultures could be differentiated into neurons, astrocytes and oligodendrocytes (Figure 4e). These spheres could be passaged and formed new spheres establishing the property of self-renewal (Figure 4f). These data indicate that there is a population of neural stem cells that is present in the spinal cord that was not detectable in embryonic cortex.

**Figure 5:**
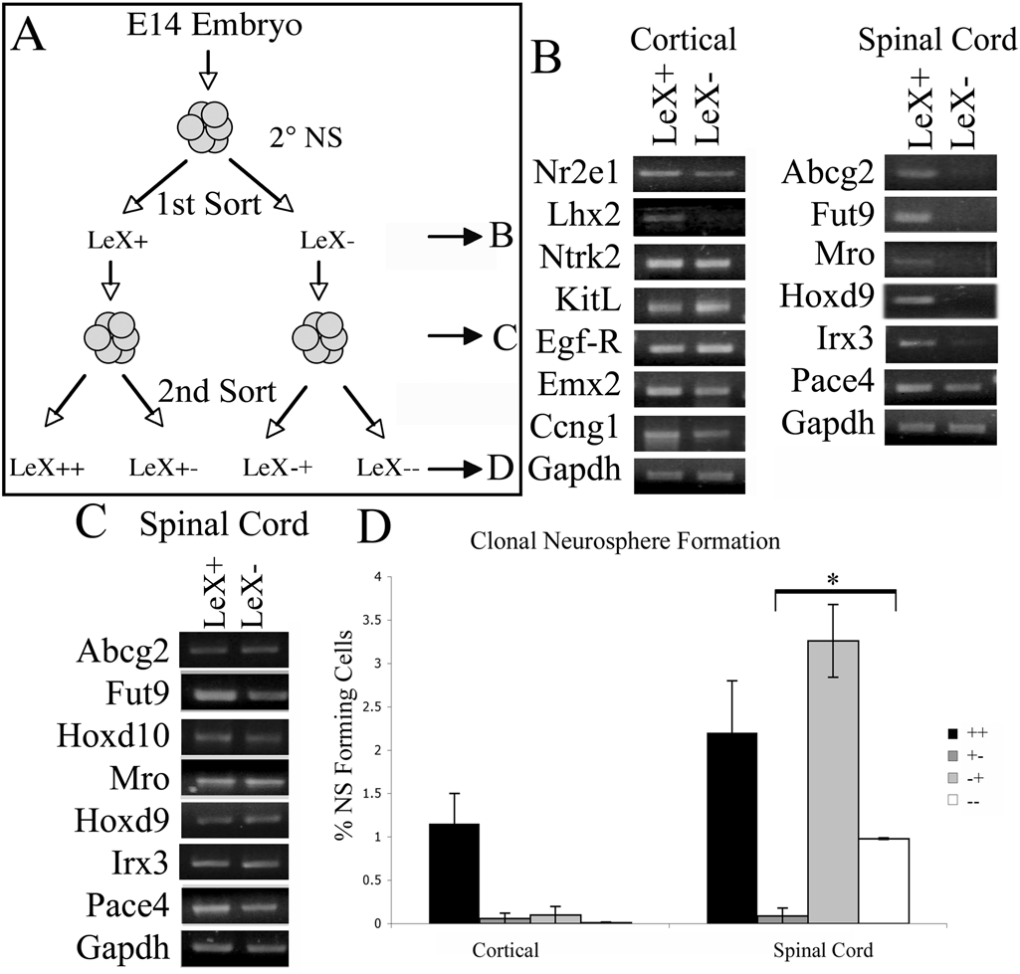
Gene Expression in LeX+ and LeX− cells derived from E14 neurospheres: (A) Schematic of double sorting protocol. B & C refer to stages at which gene expression was analyzed and D refers to clonal neurosphere forming ability of cells after two LeX sorts. (B) RT-PCR of cells immediately following first sort for LeX expression. (C) RT-PCR on neurospheres generated by LeX+ and LeX− spinal cord derived cells. (D) Clonal neurosphere forming ability of cells based on the LeX expression status at two different stages of cell culture (see panel A). First “+” or “−” refers to expression at time of 1st sort and 2nd+/−refers to second sort. * P<0.05, Anova followed by post hoc t-test.

One might hypothesize that LeX positive cells from the cortex and spinal cord would be essentially similar, while LeX negative cells would be fundamentally different, as indicated by their differential neurosphere-forming capacity. In order to test these predictions, we examined gene expression by RT-PCR. First, we examined whether genes we have associated with regional identity were housed within the LeX positive or negative fraction of cells from the embryonic cortical and spinal cord- derived neurospheres. We found that while cortical enriched genes tended to be present in both the LeX positive and LeX negative cells, all spinal cord enriched genes tested, except Pace4, were expressed exclusively by LeX positive cells (Figure 5b). Stem cell associated genes were expressed by both LeX positive and LeX negative cells (data not shown). These data indicate that LeX positive NSCs from cortical and spinal cord- derived neurospheres are not a more genetically similar progenitor population, and indicate that regional identity is not restricted only to neurosphere- forming cells. We next examined whether neurospheres generated from LeX negative spinal cord derived cells also lacked markers of regional identity. We found that neurospheres generated from LeX negative cells expressed spinal cord enriched genes and were indistinguishable from neurospheres generated from LeX positive cells (Figure 5c). These data demonstrate that while LeX negative cells do not, themselves, express markers of spinal cord identity, the neurospheres derived from these cells, do and imply that the LeX negative NSCs are regionalized in a manner not detected using the current set of markers.

We next tested the hypothesis that LeX negative cells could give rise to LeX positive NSCs. Neurospheres were generated from E14 spinal cords, sorted for LeX expression and placed back into neurosphere forming conditions (LeX+, LeX−) (1^st^ Sort, Figure 5a). Neurospheres that were generated from LeX positive and LeX negative cells were then resorted for LeX expression (LeX++. LeX+−. LeX−+. LeX−−) (2^nd^ sort, Figure 5a). We found that within spinal cord derived neurospheres, LeX positive cells derived from either LeX positive or LeX negative cells were able to form new tripotent clonal neurospheres (Figure 5d). LeX negative cells derived from LeX positive neurospheres however were not able to generate neurospheres. This implies that there is a lineage relationship between spinal cord derived LeX negative and LeX positive NSCs such that LeX negative NSCs give rise to LeX positive NSCs. In contrast, cortical derived neurospheres could only be generated from LeX positive cells (Figure 5d). The LeX negative cells that are generated by LeX positive cells are likely to be differentiated cells.

To further examine the differences between LeX positive and LeX negative NSCs we performed microarray experiments. We determined that the best comparison to identify genes that are expressed by LeX negative NSCs is to compare gene expression in neurospheres derived from LeX positive and LeX negative cells. Our rationale is that differentiated cells do not express LeX and the majority of LeX negative cells are not stem cells as demonstrated by the relative infrequency of generating neurospheres. Therefore, a direct comparison of LeX positive and LeX negative cells would result in the identification and proliferation and differentiation associated genes, respectively. We reasoned that since both LeX positive and LeX negative NSCs give rise to LeX positive NSCs and restricted progenitors, but only LeX negative NSCs can give rise to more LeX negative NSCs the only difference between neurospheres generated from LeX positive and LeX negative NSCs is the presence of LeX negative NSCs. As a result few genes would be differentially expressed between neurospheres generated from LeX positive and LeX negative NSCs. We found 73 genes were enriched 2 fold or greater in LeX positive generated neurospheres and 15 genes were enriched in LeX negative generated neurospheres (Supplemental Table 4). Future experiments are necessary to determine whether any of the 12 genes, alone or in combination, can serve as markers for LeX negative NSCs.

## Discussion

We have shown that NSCs derived from mouse embryonic cortex and spinal cord have similar proliferative abilities, but have significant differences in gene expression that are maintained in vitro and thus are likely to be cell intrinsic. We found that several genes previously implicated in NSC regulation were not differentially expressed by cortical and spinal cord derived neurospheres, suggesting that the overall genetic regulatory mechanisms of regionally distinct NSC populations is similar. Additionally, we have identified genes that are enriched in spinal cord neurospheres. Further studies can determine which of these genes are expressed specifically by multipotent NSC and which are expressed by neuronally and/or glially restricted progenitors as well as which genes are expressed by ventrally and dorsally derived neural stem and progenitors.

Surprisingly, we found two populations of spinal cord derived NSCs: one that expresses the cell surface antigen LeX and markers that are differentially expressed between spinal cord and cortical derived neurospheres; and one that does not express LeX, nor markers of regional identity. We provide evidence that these populations are lineage-related with the LeX negative NSCs giving rise to LeX positive NSCs, but not vice-versa.

### NSC behaviors are similar for cortical and spinal cord derived NSC

In this study we found that the overall stem cell characteristics of self-renewal and tripotency are similar amongst cortical and spinal cord derived NSCs. Spinal cord neural stem cells are not responsive to EGF at embryonic day 11. This inability to generate neurospheres in response to EGF alone at early developmental time points is consistent with the rostral causal gradient of EGF-R expression as shown by Rao and colleagues [41], [42]. Previous studies of brain derived NSCs have also found that bFGF responsive NSCs are present prior to EGF responsive NSCs [43], [44], [45]. In addition to comparable mitogen responsiveness, clonal neurosphere forming ability is similar between cortical and spinal cord derived NSC. Furthermore, the expression of selected genes shown to regulate or be expressed by NSCs is not different by RT-PCR or by microarray. This suggests that the overall genetic mechanisms regulating NSC behaviors are similar between cortical and spinal cord derived NSCs. The largest group of differentially expressed genes are involved in patterning, including many homeobox genes, demonstrating that patterning and NSC are closely tied together. Additionally, several differentially expressed genes are involved in sensing and responding to external environments, including genes involved in cell migration and cell adhesion suggesting that regionally distinct NSC may be apt to respond to region specific niches.

### Neural precursors maintain anterior-posterior patterning

Our studies demonstrate that some patterning persists in spinal cord-derived NSCs and suggest that there is a fundamental difference between brain and spinal cord NSCs. These findings are in seeming contradiction to some previous studies that demonstrated a lack of putative spinal cord markers in cultured progenitors. For example, one study cited a lack of detectable Hoxd1 or Hoxb9 in cultured spinal cord progenitors as evidence of the lack of regional identity [14]. However, Hoxd1 has not previously been described as expressed by spinal cord derived precursors, and Hoxb9 is expressed by 0.3% of spinal cord derived precursors, which is potentially below the limit of detection for traditional RT-PCR [46]. Furthermore hoxb9 is expressed by committed motor neuron progenitors that may not be present in undifferentiated neurospheres [47]. Therefore, selecting appropriate markers of spinal cord identity is necessary to determine whether regional identity is maintained in vitro. Here, we have presented a set of Hox genes that are enriched in spinal cord derived neurospheres that can be used in future studies as markers of regional identity.

The development of the CNS from a single sheet of neuroepithelium requires tight temporal and spatial regulation of cell type generation. Previous work by Temple and colleagues demonstrated that the temporal pattern of neurogenesis preceding gliogenesis is maintained by NSCs in vitro [48]. It may therefore not be surprising that regional identity is also cell intrinsic and maintained in vitro. The data presented here, indicate that there is a persistence of the spinal cord specific genes, Hoxc10 and Hoxd10 in serial, clonal cultures. Validating our results using clonal derived, multiply passaged neurospheres, ensures that the gene expression differences we observed were due to either direct differences in NSC gene expression or in genes expressed by NSC progeny rather than other cells that are contaminating the neurosphere cultures. These data support the hypothesis that at least some aspects of spinal cord identity are encoded in the NSC at the times examined, and point to the importance of discovering mechanisms that mediate this identity.

Not all aspects of regional identity, however, are maintained in culture, indicating that there is some plasticity in regionalization. Gabay et al. (2003) demonstrated that cultures of NSCs from either the dorsal or ventral spinal cord rapidly lose their identity in vitro, gaining markers of the other region [11]. Although we did not perform our dissections in such a way as to separate dorsal from ventral cord, when we examined gene expression in clonally derived neurospheres, we found expression of several dorsal- ventral markers, consistent with the notion that dorsoventral identity is not maintained. A lack of retention of molecular regionalization has also been described when others have examined characteristics of NSC derived from different brain regions. For example, Emx2, a forebrain-expressed homeodomain factor, is expressed ectopically in neurospheres derived from non-forebrain regions [3], [14], [49]. In addition, dorsal brain derived progenitors begin to express genes associated with ventral identity and clonal brain derived neurospheres express markers of multiple dorsoventral precursor domains [14], [50]. In addition, Hack et al. (2004) demonstrated a down-regulation of dorsal and ventral specifying transcription factors in neurospheres derived from different brain regions [15].

The maintenance of rostrocaudal, but not dorsoventral, patterning has been demonstrated in transplantation studies where lateral ganglionic eminenece derived precursors differentiated into host region (dorsoventral) specific neurons in the diencephalon and mesencephalon but continued to express Bf1, a telencephalic marker [51]. However, there is a limit to this seeming plasticity. When brain-derived progenitors are placed in culture, the homeodomain genes that they express are indicative of being brain, rather than spinal cord-derived as shown here and by others [3], [14], [49]. Furthermore, a lack of molecular regionalization, does not necessarily translate into a loss of regional identity. Horiguchi et al., (2004) showed that neurospheres derived from different brain regions, that expressed the same region specific transcription factors, had distinct proliferation rates and differentiated into neurons specific to the region from which the progenitors were isolated [52]. Thus while not all aspects of regional identity are immutable, some aspects of rostrocaudal identity are maintained by NSCs.

One potential criticism in assigning a particular gene as being cortical or spinal cord enriched is that different regions have somewhat different developmental timing sequence. One might propose that a gene that is expressed at one point in the development of an early developing region may be expressed at later times in a later developing region, and not represent true region-specificity. Therefore, in the current study, we examined gene expression in neurospheres derived from multiple developmental stages. Our data indicate that several markers of spinal cord and cortical neurospheres maintained their expression at all stages and times in culture examined. These observations suggest that the differences we observed were in fact based on the region of origin rather than different developmental process that were occurring within the brain and spinal cord at the time NSCs were isolated (or at the same embryological age).

The differences between brain and spinal cord NSCs also seem to be carried through to the tumors that they could potentially give rise to. We, and others, have isolated stem cell-like cells from CNS tumors, consistent with the hypothesis that mutations in NSCs or progenitors derived from them cause tumors [53], [54]. In an elegant study, Taylor et al., described these cancer stem cells in ependymomas [55]. Their gene expression studies demonstrated strong differences in genes, including homeodomain proteins, expressed by brain and spinal cord derived tumors. We found significant overlap between our lists of differentially expressed genes (Supplemental Table 5). Of note, we did not find any overlap between genes enriched in cortical ependymomas and spinal cord derived neurospheres nor overlap between spinal cord ependymomas and cortical derived neurospheres. Analysis of our gene expression data in normal murine NSC revealed similar sets of differentially-expressed genes, indicating both that ependymomas do likely arise from a regionally specified stem or progenitor cell and that the regional gene expression differences we observe here are likely to be of relevance to human spinal cord neural stem and progenitor cells.

### Heterogeneity amongst NSC populations

**Figure 6:**
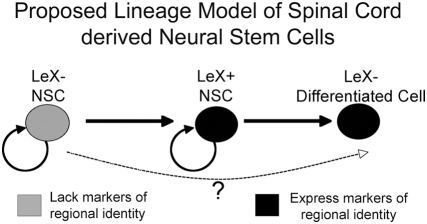
Lineage relationship of spinal cord derived NSCs. LeX− NSCs derived from the spinal cord can give rise to LeX+ and LeX− NSCs. LeX− NSCs do not express markers of regional identity while LeX+ NSCs express markers indicative of spinal cord identity. LeX− cells derived from LeX+ cells are not able to generate new clonal neurospheres and are likely differentiated cells. It is not clear whether LeX− NSC must pass through a LeX expressing stage prior to differentiation.

We were able to generate clonally passagable, tripotent neurospheres from both LeX positive and LeX negative cells. The discrimination of cells based on their expression of LeX resulted in two different cell populations. LeX positive cells express many markers of spinal cord identity, which are largely absent from LeX negative cells. We found that there is a lineage relationship between LeX negative and positive NSCs (Figure 5). LeX negative NSCs were able to self-renew as well as generate the LeX expressing NSCs. Since most differentiated cells do not express LeX, it would be expected that a LeX positive cell would give rise to both LeX negative (differentiated) and LeX positive (progenitor) cells, which is indeed the case. However, LeX negative spinal cord NSCs not only self-renew, but also give rise to LeX positive NSCs. Furthermore, although LeX negative NSCs do not express the typical spinal cord homeodomain genes, the clonal neurospheres that arise from these cells do. These data demonstrate that a LeX negative cell, one with an as yet identified spinal cord patterning program, gives rise to the LeX positive NSC population, one with explicit spinal cord properties (Figure 6). It will be important to define what mechanisms underlie this regional identity and trigger the expression of region-specific genes.

We were not able to definitively isolate the LeX negative NSC population directly from developing spinal cord tissue. We believe that this is because the majority of LeX negative cells in primary tissue are differentiated cells and the percent of LeX negative neural stem cells in primary tissue is below the limit of detection for cell sorting. The exceedingly small percentage of LeX negative NSCs likely present in primary tissue, coupled with the harshness of cell sorting has made the isolation of LeX negative NSCs in vivo problematic. The identification of additional markers to markers to enrich for this population will potentially enable verification of this population in vivo. We believe that the existence of a LeX negative NSC population is not an artifact of tissue culture as there are significant differences in the gene expression of LeX positive and negative NSC. The differences in gene expression between LeX expressing and non-expressing NSCs demonstrate that these different populations are not simply the result of instability of LeX expression. Additionally, the lineal relationship we have shown here provides further evidence of an additional stem cell population that is present in the embryonic mouse spinal cord that is not present in the brain.

### Implications for neural repair

Our observations demonstrate that there are fundamental differences between spinal cord and brain-derived NSCs and identify some of the characteristics that are specific to spinal cord NSCs. This raises the possibility that spinal cord NSCs possess information that would make them more likely to produce spinal cord appropriate cell types and therefore be more likely to successfully replace cells and/or integrate into damaged host spinal cord. Furthermore, we do not yet understand the implications of the discovery of the different, but related LeX negative and LeX positive NSCs. It is possible that the LeX negative NSCs will be less restricted and differentiate into a broader range of cell types or be more expandable in culture, thus making them more useful for repair. Our studies also will serve as a springboard to identify genes and pathways that regulate spinal cord NSC proliferation and differentiation—pathways that may be different from those utilized by brain NSCs. This understanding may allow for enhanced production of spinal cord NSCs from pluripotent cells, such as embryonic stem cells, as well as an improved ability to stimulate repair from endogenous NSCs following spinal cord injury.

## Methods

### Neural progenitor cultures

Timed pregnant and postnatal mice were obtained from Charles River Laboratories (Boston, MA). Neurosphere cultures were prepared as described previously [20]. Briefly, cortices and spinal cords were removed and dissociated with a fire-polished glass pipette, passed through a 40 µm mesh filter (Falcon) and resuspended at 50,000 cells/ml in DME/Ham's F12 medium (Invitrogen, San Diego, CA) supplemented with B27 (Invitrogen), penicillin/streptomycin (Invitrogen) and 5 µg/ml heparin (Sigma-Aldrich, St. Louis, MO), 20 ng/ml bFGF (Peprotech, Rocky Hill, NJ) and/or 50 ng/ml EGF (Peprotech). Growth factors (EGF, bFGF) were added twice per week. After 7 days in culture, neurospheres were dissociated into single cells using Trypsin-EDTA (Invitrogen) and resuspended at 50,000 cells/ml in the above media or at clonal density (1,000 cells/ml) in Neurobasal medium (Invitrogen) supplemented with B27, penicillin/streptomycin, 200 µM L-Glutamine, 2 µg/ml heparin, 20 ng/ml bFGF and 50 ng/ml EGF (modified from [56]. For differentiation, neurospheres were resuspended in Neurobasal medium supplemented with B27, penicillin/streptomycin, 40 µM L-Glutamine and 25 mM Glutamic Acid (Sigma-Aldrich) and plated on poly-L-lysine (Sigma-Aldrich) coated (10 µg/ml) coverslips for 5 days. Conditioned media were generated by culturing E14 progenitors at 50,000 cells/ml for 1 week, after which cultures were centrifuged, media removed and filtered to eliminate any remaining cells or debris and the media was frozen in single use aliquots.

### Semiquantitative RT-PCR

Total RNA was isolated from each sample using Trizol (Invitrogen), and 1 µg RNA was treated with DNAse 1 (Sigma) for 15 minutes at room temperature and converted to cDNA by reverse transcriptase following the manufacturer's protocol (Improm2; Promega, Madison, WI). For semiquantitative RT-PCR, primers for glyceraldehyde-3-phosphate-dehydrogenase gene (GAPDH) were used as an internal control from 20 to 25 cycles. After correction for GAPDH signal for each set, the resultant cDNA was subjected to PCR analysis using gene-specific primers listed in Supplemental Table 1. The following PCR conditions were used: 94° C for 2 min, followed by cycles of 94°C (1 min), 58°C or 60°C (1 min), and 72°C (1 min), with the reaction terminated by a final 2 minute incubation at 72°C. Control experiments were done either without reverse transcriptase or without template cDNA to ensure that the results were not due to amplification of genomic or contaminating DNA (not shown). Each reaction was visualized using ethidium bromide after 1.5% agarose gel electrophoresis for 30 min, and expression levels were compared between the cDNA samples on the same gel.

### Quantitative PCR

RNA extraction and cDNA synthesis was performed as described for semiquantitative PCR. The qRTPCR was done by ABI PRISM 7700 sequence detection system (Applied Biosystems, Foster City, CA) using Power SYBR Master Mix (Applied Biosystems). Thermal cycling consisted of an initial step at 95°C for 10 minutes followed by 40 cycles of 95°C for 15 seconds and 60°C for 1 minute. Analysis was done as described by Applied Biosystems (www.appliedbiosystems.com). Briefly, the delta ct for each sample was calculated as the difference between the critical threshold of the gene of interest and the critical threshold of GAPDH. The delta delta ct was calculated as the difference in the delta ct for two cDNA samples. Results are displayed as the delta delta ct of each gene in spinal cord derived neurospheres divided by the delta delta ct of the same gene in cortical derived neurospheres.

### Immunocytochemistry

Immunocytochemistry was performed as described previously [20], [57] using the following antibodies: LeX (CD15; 1∶200; CAMFoilio, Becton Dickinson, Franklin Lakes, NJ), TuJ1 (1∶500; Covance, Princeton, NJ), GFAP (1∶1,000; Dako Cytomation, Fort Collins, CO), and O4 (1∶50; Chemicon, Temecula, CA). Primary antibodies were visualized with Alexa 568– (red), 488– (green), and 350 (blue)–conjugated secondary antibodies (Invitrogen). DAPI (blue) or propridium iodide (red) was used as a fluorescent nuclear counterstain. Cells were incubated in primary antibodies overnight at 4°C and in secondary antibodies at room temperature for 1 hour.

### Fluorescent Activated Cell Sorting

Flow sorting of cells from E14 cortical and spinal cord derived neurospheres was performed with a FACSVantage (Becton Dickinson) using a purification-mode algorithm. Gating parameters were set by side and forward scatter to eliminate dead and aggregated cells. Cells were immunostianed using LeX antibody [35] for 1 hour and Alexa 488 for 1 hour. Background signals were determined by incubation of the same cells without primary antibody. After sorting, cells were either recultured in Neurobasal based neurosphere media or RNA was collected for gene expression analysis. Sorting efficiency was verified to be >98% by microscopy or by FACS (not shown).

### Microarrays and data analysis

RNA was collected from secondary neurospheres (two weeks in vitro) derived from E14 cortex and spinal cord. RNA was amplified and labeled using low input linear amplification (Agilent, Santa Clara, CA). Three sets of cultures (biological replicates) were labeled with Cy3 and Cy5 and hybridized onto Whole Mouse Genome Oligonucleotide Microarrays (Agilent) containing 41,174 probes representing 20,163 genes [58]. Each comparison was performed twice with a dye flip to control for a potential dye bias resulting in a total of 6 arrays. Arrays were washed and scanned using Agilent Bioanalyzer, signal intensities were determined using the Agilent feature extraction software and raw data transferred to Microsoft Excel and TM4 MEV microarray data analysis software (TIGR; http://www.tm4.org/mev.html; [59] for identification of differentially expressed genes. Enriched genes were identified using criteria similar to Lobo et al., 2006[60]. Briefly, enriched genes had a 1.5-fold difference in expression in each comparison, with an average ratio of 2 fold difference and significance of differential expression of P<0.01, (paired t-test) as determined by the TM4 MEV. Gene Ontology analysis was performed using DAVID/EASE (http://david.niaid.nih.gov/david/ease.htm). Genebank accession numbers were used to search for over-represented biological processes against whole mouse genome. Only categories with EASE score <0.01 were considered as significantly enriched [27], [61].

## Supporting Information

Table S1 List of PCR Primers
(0.04 MB DOC)

Table S2 List of differentially expressed genes. Criteria for differential expression>2 fold change in expression in each hybridization with P<0.01. Expression>1: enriched in spinal cord derived neurospheres.
(0.33 MB DOC)

Table S3 qRT-PCR of genes in freshly isolated cortical and spinal cord tissue. Results represent the delta delta critical threshold of cortical tissue compared to spinal cord tissue. The first seven genes were identified by microarray as enriched in cortical derived neurospheres. The bottom eight genes were identified by microarray as enriched in spinal cord derived neurospheres. Relative expression>1 indicates greater expression in spinal cord tissue.
(0.03 MB DOC)

Table S4 List of genes enriched in neuropshere derived from LeX+ cells (LeX+/LeX−>2) and genes enriched in neurospheres derived from LeX− cells (LeX+/LeX−<0.5).
(0.14 MB DOC)

Table S5 Overlap of genes in regionally specific neurospheres and ependymomas. (SC: spinal cord; ST: supratentoral). Ependymoma enriched genes identified by Taylor MD, Poppleton H, Fuller C, Su X, Liu Y, Jensen P, Magdaleno S, Dalton J, Calabrese C, Board J, Macdonald T, Rutka J, Guha A, Gajjar A, Curran T, Gilbertson RJ (2005) Radial glia cells are candidate stem cells of ependymoma. Cancer Cell 8:323–335.
(0.04 MB DOC)

Figure S1 The majority of cells within cortical and spinal cord derived neurospheres express LeX and/or Nestin. E14 secondary neurospheres were dissociated and stained with antibodies to LeX or Nestin. Bars are mean+SEM.
(0.44 MB TIF)

Figure S2 Expression of genes indicative of multiple dorsoventral regions is maintained in vitro. Gene expression was examined in primary tissue and secondary spinal cord derived neurospheres cultured at high density. Schematic represents composite of gene expression patterns throughout embryonic development, not any particular embryonic age. Genes were assessed between 25–35 cycles. Gapdh was assessed at 18 cycles.
(1.55 MB TIF)

Figure S3 DAVID gene ontology analysis reveals global changes in biological processes between cortical and spinal cord derived neural progenitors. Most significant changes are observed generally in the development category and more specifically in pattern specification, nervous system development and neurogenesis. X axis is the percentage of genes that fit within a given functional category.
(0.80 MB TIF)

## References

[1] Pluchino S, Zanotti L, Deleidi M, Martino G (2005). Neural stem cells and their use as therapeutic tool in neurological disorders.. Brain Res Brain Res Rev.

[2] Kornblum HI, Geschwind DH (2001). Molecular markers in CNS stem cell research: hitting a moving target.. Nat Rev Neurosci.

[3] Hitoshi S, Tropepe V, Ekker M, van der Kooy D (2002). Neural stem cell lineages are regionally specified, but not committed, within distinct compartments of the developing brain.. Development.

[4] Ostenfeld T, Joly E, Tai YT, Peters A, Caldwell M (2002). Regional specification of rodent and human neurospheres.. Brain Res Dev Brain Res.

[5] Watanabe K, Nakamura M, Iwanami A, Fujita Y, Kanemura Y (2004). Comparison between fetal spinal-cord- and forebrain-derived neural stem/progenitor cells as a source of transplantation for spinal cord injury.. Dev Neurosci.

[6] Weiss S, Dunne C, Hewson J, Wohl C, Wheatley M (1996). Multipotent CNS stem cells are present in the adult mammalian spinal cord and ventricular neuroaxis.. J Neurosci.

[7] Zappone MV, Galli R, Catena R, Meani N, De Biasi S (2000). Sox2 regulatory sequences direct expression of a (beta)-geo transgene to telencephalic neural stem cells and precursors of the mouse embryo, revealing regionalization of gene expression in CNS stem cells.. Development.

[8] Kim HT, Kim IS, Lee IS, Lee JP, Snyder EY (2006). Human neurospheres derived from the fetal central nervous system are regionally and temporally specified but are not committed.. Exp Neurol.

[9] He W, Ingraham C, Rising L, Goderie S, Temple S (2001). Multipotent stem cells from the mouse basal forebrain contribute GABAergic neurons and oligodendrocytes to the cerebral cortex during embryogenesis.. J Neurosci.

[10] Shihabuddin LS, Ray J, Gage FH (1997). FGF-2 is sufficient to isolate progenitors found in the adult mammalian spinal cord.. Exp Neurol.

[11] Gabay L, Lowell S, Rubin LL, Anderson DJ (2003). Deregulation of dorsoventral patterning by FGF confers trilineage differentiation capacity on CNS stem cells in vitro.. Neuron.

[12] Brock SC, Bonsall J, Luskin MB (1998). The neuronal progenitor cells of the forebrain subventricular zone: intrinsic properties in vitro and following transplantation.. Methods.

[13] Yang H, Mujtaba T, Venkatraman G, Wu YY, Rao MS (2000). Region-specific differentiation of neural tube-derived neuronal restricted progenitor cells after heterotopic transplantation.. Proc Natl Acad Sci U S A.

[14] Santa-Olalla J, Baizabal JM, Fregoso M, del Carmen Cardenas M, Covarrubias L (2003). The in vivo positional identity gene expression code is not preserved in neural stem cells grown in culture.. Eur J Neurosci.

[15] Hack MA, Sugimori M, Lundberg C, Nakafuku M, Gotz M (2004). Regionalization and fate specification in neurospheres: the role of Olig2 and Pax6.. Mol Cell Neurosci.

[16] Graham V, Khudyakov J, Ellis P, Pevny L (2003). SOX2 functions to maintain neural progenitor identity.. Neuron.

[17] Molofsky AV, Pardal R, Iwashita T, Park IK, Clarke MF (2003). Bmi-1 dependence distinguishes neural stem cell self-renewal from progenitor proliferation.. Nature.

[18] Lendahl U, Zimmerman LB, McKay RD (1990). CNS stem cells express a new class of intermediate filament protein.. Cell.

[19] Sakakibara S, Imai T, Hamaguchi K, Okabe M, Aruga J (1996). Mouse-Musashi-1, a neural RNA-binding protein highly enriched in the mammalian CNS stem cell.. Dev Biol.

[20] Geschwind DH, Ou J, Easterday MC, Dougherty JD, Jackson RL (2001). A genetic analysis of neural progenitor differentiation.. Neuron.

[21] Tsai RY, McKay RD (2002). A nucleolar mechanism controlling cell proliferation in stem cells and cancer cells.. Genes Dev.

[22] Simeone A, Gulisano M, Acampora D, Stornaiuolo A, Rambaldi M (1992). Two vertebrate homeobox genes related to the Drosophila empty spiracles gene are expressed in the embryonic cerebral cortex.. Embo J.

[23] Kimura J, Suda Y, Kurokawa D, Hossain ZM, Nakamura M (2005). Emx2 and Pax6 function in cooperation with Otx2 and Otx1 to develop caudal forebrain primordium that includes future archipallium.. J Neurosci.

[24] Herault Y, Beckers J, Kondo T, Fraudeau N, Duboule D (1998). Genetic analysis of a Hoxd-12 regulatory element reveals global versus local modes of controls in the HoxD complex.. Development.

[25] Kukekov VG, Laywell ED, Thomas LB, Steindler DA (1997). A nestin-negative precursor cell from the adult mouse brain gives rise to neurons and glia.. Glia.

[26] Nakano I, Paucar AA, Bajpai R, Dougherty JD, Zewail A (2005). Maternal embryonic leucine zipper kinase (MELK) regulates multipotent neural progenitor proliferation.. J Cell Biol.

[27] Dennis C (2003). Draft guidelines ease restrictions on use of genome sequence data.. Nature.

[28] Carpenter EM (2002). Hox genes and spinal cord development.. Dev Neurosci.

[29] Roy K, Kuznicki K, Wu Q, Sun Z, Bock D (2004). The Tlx gene regulates the timing of neurogenesis in the cortex.. J Neurosci.

[30] Shi Y, Chichung Lie D, Taupin P, Nakashima K, Ray J (2004). Expression and function of orphan nuclear receptor TLX in adult neural stem cells.. Nature.

[31] Bishop KM, Goudreau G, O'Leary DD (2000). Regulation of area identity in the mammalian neocortex by Emx2 and Pax6.. Science.

[32] Tao W, Lai E (1992). Telencephalon-restricted expression of BF-1, a new member of the HNF-3/fork head gene family, in the developing rat brain.. Neuron.

[33] Nornes HO, Das GD (1974). Temporal pattern of neurogenesis in spinal cord of rat. I. An autoradiographic study–time and sites of origin and migration and settling patterns of neuroblasts.. Brain Res.

[34] Nornes HO, Das GD (1972). Temporal pattern of neurogenesis in spinal cord: cytoarchitecture and directed growth of axons.. Proc Natl Acad Sci U S A.

[35] Capela A, Temple S (2002). LeX/ssea-1 is expressed by adult mouse CNS stem cells, identifying them as nonependymal.. Neuron.

[36] Capela A, Temple S (2006). LeX is expressed by principle progenitor cells in the embryonic nervous system, is secreted into their environment and binds Wnt-1.. Dev Biol.

[37] Bartsch D, Mai JK (1991). Distribution of the 3-fucosyl-N-acetyl-lactosamine (FAL) epitope in the adult mouse brain.. Cell Tissue Res.

[38] Ashwell KW, Mai JK (1997). Developmental expression of the CD15-epitope in the brainstem and spinal cord of the mouse.. Anat Embryol (Berl).

[39] Kim M, Morshead CM (2003). Distinct populations of forebrain neural stem and progenitor cells can be isolated using side-population analysis.. J Neurosci.

[40] Imura T, Nakano I, Kornblum HI, Sofroniew MV (2006). Phenotypic and functional heterogeneity of GFAP-expressing cells in vitro: differential expression of LeX/CD15 by GFAP-expressing multipotent neural stem cells and non-neurogenic astrocytes.. Glia.

[41] Kalyani AJ, Mujtaba T, Rao MS (1999). Expression of EGF receptor and FGF receptor isoforms during neuroepithelial stem cell differentiation.. J Neurobiol.

[42] Kalyani A, Hobson K, Rao MS (1997). Neuroepithelial stem cells from the embryonic spinal cord: isolation, characterization, and clonal analysis.. Dev Biol.

[43] Ciccolini F, Svendsen CN (1998). Fibroblast growth factor 2 (FGF-2) promotes acquisition of epidermal growth factor (EGF) responsiveness in mouse striatal precursor cells: identification of neural precursors responding to both EGF and FGF-2.. J Neurosci.

[44] Tropepe V, Sibilia M, Ciruna BG, Rossant J, Wagner EF (1999). Distinct neural stem cells proliferate in response to EGF and FGF in the developing mouse telencephalon.. Dev Biol.

[45] Irvin DK, Dhaka A, Hicks C, Weinmaster G, Kornblum HI (2003). Extrinsic and intrinsic factors governing cell fate in cortical progenitor cultures.. Dev Neurosci.

[46] Yamamoto S, Yamamoto N, Kitamura T, Nakamura K, Nakafuku M (2001). Proliferation of parenchymal neural progenitors in response to injury in the adult rat spinal cord.. Exp Neurol.

[47] Tanabe Y, Jessell TM (1996). Diversity and pattern in the developing spinal cord.. Science.

[48] Qian X, Goderie SK, Shen Q, Stern JH, Temple S (1998). Intrinsic programs of patterned cell lineages in isolated vertebrate CNS ventricular zone cells.. Development.

[49] Parmar M, Skogh C, Bjorklund A, Campbell K (2002). Regional specification of neurosphere cultures derived from subregions of the embryonic telencephalon.. Mol Cell Neurosci.

[50] Machon O, Backman M, Krauss S, Kozmik Z (2005). The cellular fate of cortical progenitors is not maintained in neurosphere cultures.. Mol Cell Neurosci.

[51] Na E, McCarthy M, Neyt C, Lai E, Fishell G (1998). Telencephalic progenitors maintain anteroposterior identities cell autonomously.. Curr Biol.

[52] Horiguchi S, Takahashi J, Kishi Y, Morizane A, Okamoto Y (2004). Neural precursor cells derived from human embryonic brain retain regional specificity.. J Neurosci Res.

[53] Hemmati HD, Nakano I, Lazareff JA, Masterman-Smith M, Geschwind DH (2003). Cancerous stem cells can arise from pediatric brain tumors.. Proc Natl Acad Sci U S A.

[54] Singh SK, Clarke ID, Terasaki M, Bonn VE, Hawkins C (2003). Identification of a cancer stem cell in human brain tumors.. Cancer Res.

[55] Taylor MD, Poppleton H, Fuller C, Su X, Liu Y (2005). Radial glia cells are candidate stem cells of ependymoma.. Cancer Cell.

[56] Wachs FP, Couillard-Despres S, Engelhardt M, Wilhelm D, Ploetz S (2003). High efficacy of clonal growth and expansion of adult neural stem cells.. Lab Invest.

[57] Kornblum HI, Hussain R, Wiesen J, Miettinen P, Zurcher SD (1998). Abnormal astrocyte development and neuronal death in mice lacking the epidermal growth factor receptor.. J Neurosci Res.

[58] Verdugo RA, Medrano JF (2006). Comparison of gene coverage of mouse oligonucleotide microarray platforms.. BMC Genomics.

[59] Saeed AI, Sharov V, White J, Li J, Liang W (2003). TM4: a free, open-source system for microarray data management and analysis.. Biotechniques.

[60] Lobo MK, Karsten SL, Gray M, Geschwind DH, Yang XW (2006). FACS-array profiling of striatal projection neuron subtypes in juvenile and adult mouse brains.. Nat Neurosci.

[61] Karsten SL, Kudo LC, Jackson R, Sabatti C, Kornblum HI (2003). Global analysis of gene expression in neural progenitors reveals specific cell-cycle, signaling, and metabolic networks.. Dev Biol.

