# Supplementary material for: Cell Lineage and Regional Identity of Cultured Spinal Cord Neural Stem Cells and Comparison to Brain-Derived Neural Stem Cells
Source: PLoS One. 2009 Jan 16;4(1):e4213. doi: 10.1371/journal.pone.0004213 (PMC2615219; doi:10.1371/journal.pone.0004213)
Supplement: Table S2 — List of differentially expressed genes. Criteria for differential expression>2 fold change in expression in each hybridization with P<0.01. Expression>1: enriched in spinal cord derived neurospheres. (0.33 MB DOC) [file pone.0004213.s002.doc]

| Spinal Cord Enriched Genes |  |  |  |  |
| --- | --- | --- | --- | --- |
| Gene Name | Accession Number | Description | Spinal Cord/ Cortical | P-Value |
| Hoxc9 | NM_008272 | homeo box C9 (Hoxc9) | 68.981 | 0.003 |
| Hoxb3 | NM_010458, BB807385 | homeo box B3 (Hoxb3) | 32.45 | 0.007 |
| Hoxb7 | NM_010460 | homeo box B7 (Hoxb7) | 24.452 | 0.004 |
| Hoxa7 | NM_010455 | homeo box A7 (Hoxa7) | 20.379 | 0.003 |
| Hoxb2 | NM_134032 | homeo box B2 (Hoxb2) | 18.731 | 0.002 |
| Hoxc6 | NM_010465 | homeo box C6 (Hoxc6) | 17.638 | 0.002 |
| Hoxa5 | NM_010453 | homeo box A5 (Hoxa5) | 16.475 | 0.007 |
| Hoxb6 | NM_008269 | homeo box B6 (Hoxb6) | 15.655 | 0.001 |
| Hoxa9 | NM_010456 | homeo box A9 (Hoxa9) | 13.163 | 0 |
| Foxb1 | NM_022378 | forkhead box B1 (Foxb1) | 11.952 | 0.004 |
| Nr2f2 | NM_009697, X76653 | nuclear receptor subfamily 2, group F, member 2 (Nr2f2) | 11.174 | 0 |
| Gabpb2 | NM_030725 | GA repeat binding protein, beta 2 | 10.303 | 0.005 |
| Hoxb9 | NM_008270, AK078860 | homeo box B9 (Hoxb9) | 10.1 | 0.002 |
| Runx2 | D14636 | Mouse mRNA for PEBP2a1 protein | 7.776 | 0.003 |
| Calca | NM_007587 | calcitonin/calcitonin-related polypeptide, alpha (Calca) | 7.209 | 0 |
| Irx1 | TC1244369 | Iroquois-class homeodomain protein IRX-1 | 6.672 | 0.007 |
| Itga9 | NM_133721 | integrin alpha 9 (Itga9) | 6.463 | 0 |
| Glis1 | NM_147221 | GLIS family zinc finger 1 (Glis1) | 6.381 | 0.002 |
| Grid2 | AK032669, AK036230, AK036246, AK034271 | Glutamate receptor, ionotropic, delta 2 | 5.655 | 0.003 |
| Kcne1l | NM_021487 | potassium voltage-gated channel, Isk-related family, member 1-like (Kcne1l) | 6.06 | 0.003 |
| Cdc42ep3 | NM_026514 | CDC42 effector protein (Rho GTPase binding) 3 (Cdc42ep3) | 5.4 | 0 |
| Gpr49 | NM_010195 | G protein-coupled receptor 49 (Gpr49) | 5.324 | 0.004 |
| Hoxc10 | NM_010462 | homeo box C10 (Hoxc10) | 5.221 | 0.002 |
| Pace4 | BC037450 | paired basic amino acid cleaving system 4 | 5.212 | 0 |
| Irx2 | NM_010574 | Iroquois related homeobox 2 (Drosophila) (Irx2) | 4.95 | 0.003 |
| Cav1 | NM_007616 | caveolin, caveolae protein 1 (Cav1) | 4.867 | 0.009 |
| Scap2 | NM_018773 | src family associated phosphoprotein 2 (Scap2) | 4.864 | 0.001 |
| 3110035E14Rik | NM_178399 | RIKEN cDNA 3110035E14 gene | 4.752 | 0.008 |
| Gabra4 | NM_010251 | gamma-aminobutyric acid (GABA-A) receptor, subunit alpha 4 (Gabra4) | 4.706 | 0.004 |
| D1Ertd471e | BC035277 | Mus musculus DNA segment, Chr 1, ERATO Doi 471, expressed, mRNA (cDNA clone IMAGE:5064213), partial cds | 4.656 | 0.003 |
| AK013705 | AK013705 | RIKEN cDNA 4922502B01 gene | 4.456 | 0 |
| AI854517 | XM_489189 | PREDICTED: Mus musculus expressed sequence AI854517 (AI854517) | 4.384 | 0 |
| 1700013E18Rik | NM_175176 | RIKEN cDNA 1700013E18 gene | 4.379 | 0.005 |
| Spon1 | NM_145584, AK084717 | spondin 1, (f-spondin) extracellular matrix protein (Spon1) | 4 | 0.001 |
| Cpne8 | NM_025815 | copine VIII (Cpne8) | 4 | 0.004 |
| Slc24a3 | NM_053195 | solute carrier family 24 (sodium/potassium/calcium exchanger), member 3 (Slc24a3) | 3.951 | 0.003 |
| Hoxd9 | NM_013555 | homeo box D9 (Hoxd9) | 3.8 | 0 |
| 2610024M03Rik | AK011539 | Protein phosphatase 1, regulatory subunit 3D | 3.738 | 0.001 |
| Six1 | NM_009189, X80339 | sine oculis-related homeobox 1 homolog (Drosophila) (Six1) | 3.657 | 0.006 |
| Boc | NM_172506 | biregional cell adhesion molecule-related/down-regulated by oncogenes (Cdon) binding protein (Boc) | 3.648 | 0.007 |
| AK083952 | AK083952 | Mus musculus 12 days embryo spinal ganglion cDNA, RIKEN full-length enriched library, clone:D130069E17 product:unknown EST, full insert sequence | 3.534 | 0.008 |
| Pkp2 | NM_026163 | plakophilin 2 (Pkp2) | 3.497 | 0.009 |
| Itga9 | AK032822 | Integrin alpha 9 (Itga9) | 3.487 | 0 |
| AW554518 | AV255740 | AV255740 RIKEN full-length enriched, adult male testis (DH10B) Mus musculus cDNA clone 4921522H04 3'. | 3.442 | 0.001 |
| Rgs8 | NM_026380 | Regulator of G-protein signaling 8 | 3.429 | 0.005 |
| AK049020 | AK049020 | Mus musculus 0 day neonate cerebellum cDNA, RIKEN full-length enriched library, clone:C230091O15 product:inferred: KIAA1276 protein {Homo sapiens}, full insert sequence. | 3.339 | 0.002 |
| Robo2 | NM_175549, AK129396, AK084163 | roundabout homolog 2 (Drosophila) (Robo2) | 3.291 | 0.007 |
| BC064078 | BF138688 | 601781871F1 NCI_CGAP_Lu30 Mus musculus cDNA clone IMAGE:4009937 5'. | 3.231 | 0.002 |
| St8sia1 | BI736113 | ST8 alpha-N-acetyl-neuraminide alpha-2,8-sialyltransferase 1 | 3.216 | 0.005 |
| Irx3 | NM_008393 | Iroquois related homeobox 3 (Drosophila) (Irx3) | 3.208 | 0.004 |
| 9330117B14 | AK033923 | Mus musculus adult male diencephalon cDNA, RIKEN full-length enriched library, clone:9330117B14 product:unknown EST, full insert sequence | 3.208 | 0 |
| Gmpr | NM_025508 | guanosine monophosphate reductase (Gmpr) | 3.153 | 0 |
| Slitrk6 | NM_175499 | SLIT and NTRK-like family, member 6 (Slitrk6) | 3.141 | 0.001 |
| Car13 | NM_024495 | carbonic anhydrase 13 (Car13) | 3.049 | 0.006 |
| 1700084M14Rik | CN833688 | AGENCOURT_24954556 NIH_MGC_169 Mus musculus cDNA clone IMAGE:30917828 5' sequence | 2.995 | 0.001 |
| Gstt3 | NM_133994 | glutathione S-transferase, theta 3 (Gstt3) | 2.968 | 0 |
| Ankrd15 | NM_181404 | ankyrin repeat domain 15 (Ankrd15) | 2.963 | 0.005 |
| Kns2 | NM_008450 | kinesin 2 (Kns2) | 2.917 | 0.003 |
| Sned1 | NM_172463 | sushi, nidogen and EGF-like domains 1 (Sned1) | 2.891 | 0 |
| Gap43 | NM_008083 | growth associated protein 43 (Gap43) | 2.888 | 0.001 |
| Grb10 | BC053842, BC016111 | Growth factor receptor bound protein 10 | 2.85 | 0.006 |
| Tmem145 | NM_183311 | Transmembrane protein 145 | 2.832 | 0.002 |
| Acyp2 | NM_029344 | acylphosphatase 2, muscle type (Acyp2) | 2.831 | 0.002 |
| 2310014D11Rik | AK009333 | Mus musculus adult male tongue cDNA, RIKEN full-length enriched library, clone:2310014D11 product:unknown EST, full insert sequence. | 2.815 | 0.008 |
| AK046848 | AK046848 | Mus musculus 10 days neonate medulla oblongata cDNA, RIKEN full-length enriched library, clone:B830028K19 product:unknown EST, full insert sequence. | 2.797 | 0.002 |
| 9430028L06Rik | BC075677 | Mus musculus cDNA clone IMAGE:30619724, partial cds | 2.788 | 0.002 |
| 2310043N10Rik | AK028745 | Mus musculus 10 days neonate skin cDNA, RIKEN full-length enriched library, clone:4732452C17 product:unknown EST, full insert sequence | 2.767 | 0 |
| Pde1b | NM_008800 | phosphodiesterase 1B, Ca2+-calmodulin dependent (Pde1b) | 2.74 | 0.007 |
| Tceal1 | NM_146236 | transcription elongation factor A (SII)-like 1 (Tceal1) | 2.704 | 0.007 |
| Mfsd2 | NM_029662 | Major facilitator superfamily domain containing 2 | 2.689 | 0 |
| AI427515 | NM_173016 | Mus musculus expressed sequence AI427515 (AI427515) | 2.65 | 0.004 |
| Dleu7 | NM_173419 | Mus musculus cDNA sequence BC038059 (BC038059) | 2.634 | 0.002 |
| Atp1a1 | NM_144900 | ATPase, Na+/K+ transporting, alpha 1 polypeptide (Atp1a1) | 2.6 | 0.009 |
| AK082805 | AK082805 | Mus musculus ES cells cDNA, RIKEN full-length enriched library, clone:C330023J09 product:weakly similar to GAG POLYPROTEIN , full insert sequence. | 2.598 | 0 |
| Usmg4 | NM_031401 | upregulated during skeletal muscle growth 4 (Usmg4) | 2.588 | 0.001 |
| Unc5a | NM_153131 | unc-5 homolog A (C. elegans) (Unc5a) | 2.586 | 0 |
| Pcdha6 | NM_007767 | protocadherin alpha 6 (Pcdha6) | 2.529 | 0.001 |
| 4933436C20Rik | AK017076 | Mus musculus adult male testis cDNA, RIKEN full-length enriched library, clone:4933436C20 product:hypothetical protein, full insert sequence. | 2.512 | 0.002 |
| Enpp1 | NM_008813 | ectonucleotide pyrophosphatase/phosphodiesterase 1 (Enpp1) | 2.501 | 0.001 |
| Mmp16 | AK034828 | Matrix metallopeptidase 16 | 2.492 | 0.006 |
| Gpr45 | NM_053107 | G protein-coupled receptor 45 (Gpr45) | 2.479 | 0 |
| Tjp2 | NM_011597 | tight junction protein 2 (Tjp2) | 2.451 | 0.001 |
| Abcg2 | NM_011920 | ATP-binding cassette, sub-family G (WHITE), member 2 (Abcg2) | 2.45 | 0 |
| Stk33 | XM_358897 | PREDICTED: Mus musculus serine | 2.448 | 0 |
| Gabrg1 | NM_010252 | gamma-aminobutyric acid (GABA-A) receptor, subunit gamma 1 (Gabrg1) | 2.444 | 0 |
| 2810031P15Rik | XM_143826 | MKIAA1952 protein; PREDICTED: Mus musculus RIKEN cDNA 2810031P15 gene | 2.437 | 0 |
| Acvrinp1 | NM_015823 | activin receptor interacting protein 1 (Acvrinp1) | 2.407 | 0.006 |
| Abca7 | NM_013850 | ATP-binding cassette, sub-family A (ABC1), member 7 (Abca7) | 2.404 | 0.01 |
| Imp2 | NM_183029 | Insulin-like growth factor 2 mRNA-binding protein 2 | 2.403 | 0.001 |
| Cav2 | NM_016900 | caveolin 2 (Cav2) | 2.4 | 0.003 |
| A930031L14Rik | XM_485569 | PREDICTED: Mus musculus RIKEN cDNA A930031L14 gene | 2.396 | 0 |
| E030049G20Rik | NM_172484 | Mus musculus RIKEN cDNA E030049G20 gene | 2.393 | 0.004 |
| Bmp3 | NM_173404 | bone morphogenetic protein 3 (Bmp3) | 2.373 | 0.003 |
| Reln | NM_011261 | reelin (Reln) | 2.354 | 0.005 |
| A930041G11Rik | NM_177033 | Mus musculus RIKEN cDNA A930041G11 gene | 2.324 | 0.004 |
| Gnb4 | NM_013531 | guanine nucleotide binding protein, beta 4 (Gnb4) | 2.322 | 0.001 |
| Rcor2 changed | NM_054048 | MAP/microtubule affinity-regulating kinase 2 | 2.299 | 0.004 |
| AY425619 | AY425619 | RAMP4-2 mRNA | 2.289 | 0 |
| Sipa1l1 | NM_172579, AK122284, AK043743 | signal-induced proliferation-associated 1 like 1 (Sipa1l1) | 2.286 | 0.001 |
| Tmem28 | NM_173446 | Transmembrane protein 28 (Tmem28) | 2.271 | 0 |
| FGFR5beta | NM_054071 | fibroblast growth factor receptor-like 1 (Fgfrl1) | 2.267 | 0.007 |
| Cbln2 | NM_172633 | cerebellin 2 precursor protein (Cbln2) | 2.264 | 0.001 |
| BB254136 | BB254136 | BB254136 RIKEN full-length enriched, 7 days neonate cerebellum Mus musculus cDNA clone A730060K06 3'. | 2.26 | 0.001 |
| Lama4 | NM_010681 | laminin, alpha 4 (Lama4) | 2.25 | 0.006 |
| Dpysl4 | NM_011993 | dihydropyrimidinase-like 4 (Dpysl4) | 2.248 | 0 |
| Odz3 | NM_011857, AK031268 | odd Oz/ten-m homolog 3 (Drosophila) (Odz3) | 2.246 | 0.007 |
| Rnd1 | NM_172612 | Rho family GTPase 1 (Rnd1) | 2.241 | 0.007 |
| Ppp3r2 | AK032394 | Protein phosphatase 3, regulatory subunit B, alpha isoform (calcineurin B, type II) | 2.24 | 0.008 |
| AB024689 | AB024689 | Mus musculus gene, exon 3, partial sequence | 2.229 | 0.001 |
| Fut9 | NM_010243 | fucosyltransferase 9 (Fut9) | 2.225 | 0.001 |
| Cdkl5 | AK052380 | Gap junction membrane channel protein alpha 6 | 2.218 | 0.001 |
| Pcdh9 | BC060736 | Protocadherin 9 | 2.213 | 0.005 |
| 6430517E21Rik | NM_207583 | Mus musculus RIKEN cDNA 6430517E21 gene | 2.211 | 0 |
| Nod1 | NM_172729 | Caspase recruitment domain 4 | 2.206 | 0.006 |
| D430019H16Rik | BC058677 | Mus musculus RIKEN cDNA D430019H16 gene, mRNA (cDNA clone IMAGE:6834465), partial cds | 2.196 | 0 |
| 2900074C18Rik | AK013779 | Tetratricopeptide repeat domain 9B | 2.194 | 0 |
| Dll3 | NM_007866 | Delta-like 3 (Drosophila) | 2.194 | 0.005 |
| Nrarp | NM_025980 | Notch-regulated ankyrin repeat protein (Nrarp) | 2.189 | 0.001 |
| Lphn3 | AK122367 | Latrophilin 3 | 2.175 | 0.001 |
| 9430023B20Rik | NM_177595 | Mohawk | 2.151 | 0 |
| Dcc | AK048768 | Deleted in colorectal carcinoma (Dcc) | 2.144 | 0 |
| B830045N13Rik | NM_153539 | Mus musculus RIKEN cDNA B830045N13 gene | 2.14 | 0 |
| Rgnef | NM_012026 | Rho-guanine nucleotide exchange factor (Rgnef) | 2.14 | 0 |
| Pcdha9 | NM_138661 | protocadherin alpha 9 (Pcdha9) | 2.137 | 0.001 |
| 2900041A09Rik | NM_182839 | Mus musculus RIKEN cDNA 2900041A09 gene | 2.129 | 0.003 |
| BC030477 | NM_177618 | MKIAA0523 protein; Mus musculus cDNA sequence BC030477 (BC030477) | 2.123 | 0.001 |
| MGC67174 | NM_001004180 | Mus musculus similar to hypothetical protein FLJ14721 (MGC67174) | 2.113 | 0.003 |
| Timp2 | NM_011594 | tissue inhibitor of metalloproteinase 2 (Timp2) | 2.112 | 0.001 |
| Pgcp | NM_018755 | plasma glutamate carboxypeptidase (Pgcp) | 2.102 | 0.001 |
| C430049B03Rik | XM_489350 | PREDICTED: Mus musculus RIKEN cDNA 2700063P19 gene | 2.095 | 0.001 |
| Ogfrl1 | BC019747 | Opioid growth factor receptor-like 1 | 2.095 | 0.009 |
| Gstt1 | NM_008185 | glutathione S-transferase, theta 1 (Gstt1) | 2.092 | 0 |
| NAP042792-1 | NAP042792-1 | Unknown | 2.091 | 0.004 |
| Jmjd1c | AK050573 | Jumonji domain containing 1C | 2.089 | 0.002 |
| Scrn1 | NM_027268 | secernin 1 (Scrn1) | 2.086 | 0 |
| Lix1l | AK051276 | Lix1-like | 2.073 | 0.004 |
| Hoxa4 | NM_008265 | homeo box A4 (Hoxa4) | 2.069 | 0.003 |
| Fgf13 | NM_010200 | fibroblast growth factor 13 (Fgf13) | 2.069 | 0.005 |
| Akr1c13 | NM_013778 | aldo-keto reductase family 1, member C13 (Akr1c13) | 2.051 | 0 |
| D230005D02Rik | NM_172813 | Mus musculus RIKEN cDNA D230005D02 gene | 2.051 | 0.001 |
| BC022623 | NM_177632 | Mus musculus cDNA sequence BC022623 (BC022623) | 2.035 | 0.009 |
| Fbxo7 | BC059894 | F-box only protein 7 (Fbxo7) | 2.033 | 0.002 |
| Odz2 | NM_011856 | odd Oz/ten-m homolog 2 (Drosophila) (Odz2) | 2.03 | 0 |
| Adamts5 | NM_011782 | a disintegrin-like and metalloprotease (reprolysin type) with thrombospondin type 1 motif, 5 (aggrecanase-2) (Adamts5) | 2.029 | 0.001 |
| Gadd45g | NM_011817 | growth arrest and DNA-damage-inducible 45 gamma (Gadd45g) | 2.024 | 0.004 |
| Kcnd2 | NM_019697 | potassium voltage-gated channel, Shal-related family, member 2 (Kcnd2) | 2.024 | 0.004 |
| Catna2 | NM_009819 | catenin alpha 2 (Catna2) | 2.022 | 0.002 |
| Fxyd6 | NM_022004 | FXYD domain-containing ion transport regulator 6 (Fxyd6) | 2.018 | 0 |
| Anxa2 | AK087259 | Annexin A2 | 2.306 | 0.001 |
| Mro | NM_027741 | Maestro (Mro) | 2.002 | 0.001 |
|  |  |  |  |  |
| Cortical Enriched Genes |  |  |  |  |
| Gene Name | Accession Number | Description | Spinal Cord/ Cortical | P-Value |
| Nr2e1 | NM_152229 | nuclear receptor subfamily 2, group E, member 1 (Nr2e1) | 0.019 | 0 |
| Eya1 | BC066860 | Eyes absent 1 homolog (Drosophila) | 0.122 | 0.002 |
| Nrn1 | NM_153529 | neuritin 1 (Nrn1) | 0.134 | 0.001 |
| Lhx2 | NM_010710 | LIM homeobox protein 2 (Lhx2) | 0.149 | 0 |
| Bmper | NM_028472 | BMP-binding endothelial regulator (Bmper) | 0.196 | 0.001 |
| Dscr1l1 | NM_207649 | Down syndrome critical region gene 1-like 1 (Dscr1l1), transcript variant 1 | 0.225 | 0.001 |
| AW125753 | NM_029007 | Mus musculus expressed sequence AW125753 | 0.254 | 0.005 |
| Rlbp1 | NM_020599 | retinaldehyde binding protein 1 (Rlbp1) | 0.262 | 0.002 |
| Col4a1 | NM_009931 | procollagen, type IV, alpha 1 (Col4a1) | 0.271 | 0.001 |
| Arx | NM_007492 | aristaless related homeobox gene (Drosophila) (Arx) | 0.28 | 0 |
| Thrsp | NM_009381 | thyroid hormone responsive SPOT14 homolog (Rattus) (Thrsp) | 0.288 | 0.002 |
| Thy1 | NM_009382 | thymus cell antigen 1, theta (Thy1) | 0.288 | 0.006 |
| Egfr | NM_007912 | epidermal growth factor receptor (Egfr), transcript variant 2 | 0.292 | 0.001 |
| Kitl | NM_013598 | kit ligand (Kitl) | 0.299 | 0 |
| Tcfcp2l1 | AK086294 | Transcription factor CP2-like 1 | 0.306 | 0.002 |
| Clstn2 | NM_022319 | calsyntenin 2 (Clstn2) | 0.308 | 0 |
| Ctsh | NM_007801 | cathepsin H (Ctsh) | 0.316 | 0 |
| Pdlim3 | NM_016798 | PDZ and LIM domain 3 (Pdlim3) | 0.329 | 0.003 |
| Mmp14 | NM_008608 | matrix metalloproteinase 14 (membrane-inserted) (Mmp14) | 0.336 | 0.005 |
| Efnb2 | NM_010111 | ephrin B2 (Efnb2) | 0.337 | 0.002 |
| Cd302 | NM_025422 | CD302 antigen | 0.344 | 0.004 |
| Ntng1 | AK053689 | Netrin G1 | 0.346 | 0.001 |
| Cd44 | AK028981 | CD44 antigen | 0.359 | 0.001 |
| Bcar3 | NM_013867 | breast cancer anti-estrogen resistance 3 (Bcar3) | 0.363 | 0.007 |
| Car2 | NM_009801 | carbonic anhydrase 2 (Car2) | 0.369 | 0.008 |
| Ociad2 | NM_026950 | OCIA domain containing 2 (Ociad2) | 0.37 | 0.001 |
| Cacna2d1 | NM_009784 | calcium channel, voltage-dependent, alpha2/delta subunit 1 (Cacna2d1) | 0.372 | 0.008 |
| Smc5l1 | NM_153808 | SMC5 structural maintenance of chromosomes 5-like 1 (yeast) (Smc5l1) | 0.375 | 0 |
| Nebl | AK042301 | Nebulette | 0.38 | 0 |
| Col18a1 | NM_009929 | procollagen, type XVIII, alpha 1 (Col18a1) | 0.385 | 0.006 |
| Gria2 | NM_013540 | glutamate receptor, ionotropic, AMPA2 (alpha 2) (Gria2) | 0.39 | 0 |
| BU534286 | BU534286 | BU534286 AGENCOURT_10198645 NIH_MGC_143 Mus musculus cDNA clone IMAGE:6561559 5', mRNA sequence | 0.393 | 0 |
| Perp | NM_022032 | PERP, TP53 apoptosis effector (Perp) | 0.398 | 0.002 |
| Cacng5 | NM_080644 | calcium channel, voltage-dependent, gamma subunit 5 (Cacng5) | 0.398 | 0.003 |
| PNAT3 | NM_144533 | Nicotinamide nucleotide adenylyltransferase 3 | 0.402 | 0.004 |
| Lrrc8b | AK054249 | Leucine rich repeat containing 8 family, member B | 0.402 | 0 |
| 2210408K08 | 2210408K08 | unknown EST | 0.406 | 0 |
| Glt25d2 | NM_177756 | glycosyltransferase 25 domain containing 2 (Glt25d2) | 0.41 | 0.005 |
| Dcamkl1 | NM_019978 | double cortin and calcium/calmodulin-dependent protein kinase-like 1 (Dcamkl1) | 0.41 | 0 |
| Megf6 | BC039980 | Multiple EGF-like-domains 6 | 0.412 | 0.001 |
| B3galt5 | NM_033149 | UDP-Gal:betaGlcNAc beta 1,3-galactosyltransferase, polypeptide 5 (B3galt5) | 0.413 | 0.004 |
| Tbc1d1 | NM_019636 | TBC1 domain family, member 1 (Tbc1d1) | 0.416 | 0.001 |
| Ntrk2 | NM_008745 | neurotrophic tyrosine kinase, receptor, type 2 (Ntrk2) | 0.416 | 0 |
| Apba2 | NM_007461 | Amyloid beta (A4) precursor protein-binding, family A, member 2 | 0.417 | 0.001 |
| 4732435N03Rik | NM_172753 | Mus musculus RIKEN cDNA 4732435N03 gene | 0.417 | 0.001 |
| Gchfr | NM_177157 | GTP cyclohydrolase I feedback regulator (Gchfr) | 0.417 | 0.004 |
| Tubb6 | NM_026473 | tubulin, beta 6 (Tubb6) | 0.422 | 0.009 |
| 0610040J01Rik | NM_029554 | Mus musculus RIKEN cDNA 0610040J01 gene | 0.425 | 0 |
| Cgref1 | BC023116 | Cell growth regulator with EF hand domain 1 | 0.425 | 0.001 |
| Pitpnc1 | BC082333 | Phosphatidylinositol transfer protein, cytoplasmic 1 | 0.428 | 0.001 |
| D330050I23Rik | NM_207269 | Mus musculus RIKEN cDNA D330050I23 gene | 0.434 | 0.002 |
| Pmaip1 | NM_021451 | phorbol-12-myristate-13-acetate-induced protein 1 (Pmaip1) | 0.439 | 0.005 |
| Tnfrsf19 | NM_013869 | tumor necrosis factor receptor superfamily, member 19 (Tnfrsf19) | 0.439 | 0 |
| Ptprv | NM_007955 | protein tyrosine phosphatase, receptor type, V (Ptprv) | 0.442 | 0.004 |
| Rorb | AK044421 | RAR-related orphan receptor beta (Rorb) | 0.443 | 0 |
| 9130213B05Rik | NM_145562 | Mus musculus RIKEN cDNA 9130213B05 gene | 0.445 | 0.003 |
| Ccng1 | NM_009831 | cyclin G1 (Ccng1) | 0.445 | 0.001 |
| Dapk2 | NM_010019 | death-associated kinase 2 (Dapk2) | 0.45 | 0.002 |
| 5330438E18Rik | NM_177282 | Mus musculus RIKEN cDNA 5330438E18 gene | 0.451 | 0.006 |
| Dhx32 | NM_133941 | DEAH (Asp-Glu-Ala-His) box polypeptide 32 (Dhx32) | 0.453 | 0 |
| Rnf32 | NM_021470 | ring finger protein 32 (Rnf32) | 0.456 | 0.002 |
| 1810049K24Rik | NM_030209 | LCCL domain containing cysteine-rich secretory protein 2 (Lcrisp2) | 0.459 | 0 |
| Mfge8 | NM_008594 | milk fat globule-EGF factor 8 protein (Mfge8) | 0.459 | 0.001 |
| Lynx1 | NM_011838 | Ly6/neurotoxin 1 (Lynx1) | 0.461 | 0.001 |
| Tmem38a | NM_144534 | transmembrane protein 38a (Tmem38a) | 0.465 | 0 |
| Dab1 | NM_010014 | disabled homolog 1 (Drosophila) (Dab1) | 0.466 | 0.002 |
| Satb1 | AK088459 | Special AT-rich sequence binding protein 1 | 0.467 | 0.001 |
| Cdkn1a | NM_007669 | cyclin-dependent kinase inhibitor 1A (P21) (Cdkn1a) | 0.474 | 0.001 |
| 6332401O19Rik | NM_177013 | Mus musculus RIKEN cDNA 6332401O19 gene | 0.481 | 0 |
| TC1341860 | TC1341860 | MLR_DROME (P18432) Myosin regulatory light chain 2 (MLC-2), partial (6%) | 0.483 | 0.002 |
| Gypc | NM_027863 | glycophorin C (Gypc) | 0.484 | 0.001 |
| AK086515 | AK086515 | Mus musculus 15 days embryo head cDNA, RIKEN full-length enriched library, clone:D930033J18 product:protein tyrosine phosphatase, non-receptor type 14, full insert sequence. | 0.487 | 0.006 |
| Srxn1 | NM_029688 | Sulfiredoxin 1 homolog (S. cerevisiae) | 0.49 | 0.004 |
| St8sia5 | X98014 | alpha-2,8-sialyltransferase. | 0.491 | 0 |
| Dusp10 | NM_022019 | dual specificity phosphatase 10 (Dusp10) | 0.492 | 0.002 |
| Tmem35 | NM_026239 | transmembrane protein 35 (Tmem35) | 0.494 | 0.002 |
| Nr4a2 | NM_013613 | nuclear receptor subfamily 4, group A, member 2 (Nr4a2) | 0.5 | 0.001 |
| Klf3 | NM_008453 | Kruppel-like factor 3 (basic) (Klf3) | 0.507 | 0.008 |
| Psrc1 | NM_019976 | Proline/serine-rich coiled-coil 1 | 0.519 | 0.003 |

Table S2: List of differentially expressed genes. Criteria for differential expression > 2 fold change in expression in each hybridization with P <0.01. Expression >1: enriched in spinal cord derived neurospheres.
