# Supplementary material for: Cell Lineage and Regional Identity of Cultured Spinal Cord Neural Stem Cells and Comparison to Brain-Derived Neural Stem Cells
Source: PLoS One. 2009 Jan 16;4(1):e4213. doi: 10.1371/journal.pone.0004213 (PMC2615219; doi:10.1371/journal.pone.0004213)
Supplement: Table S3 — qRT-PCR of genes in freshly isolated cortical and spinal cord tissue. Results represent the delta delta critical threshold of cortical tissue compared to spinal cord tissue. The first seven genes were identified by microarray as enriched in cortical derived neurospheres. The bottom eight genes were identified by microarray as enriched in spinal cord derived neurospheres. Relative expression>1 indicates greater expression in spinal cord tissue. (0.03 MB DOC) [file pone.0004213.s003.doc]

| Expression in Spinal Cord tissue relative to Cortical Tissue | |
| --- | --- |
| Lhx2 | 0.0233 |
| Nr2e1 | 0.0022 |
| Emx2 | 0.0114 |
| Arx | 1.2850 |
| Egf-R | 0.0011 |
| Ntrk2 | 1.4140 |
| Ccng1 | 1.0607 |
|  |  |
| Hoxd10 | 1165.3468 |
| Pcsk6 | 9.2755 |
| Irx3 | 32.7606 |
| Anxa2 | 5.4105 |
| Abcg2 | 0.7601 |
| Cav2 | 2.1658 |
| Fut9 | 0.5253 |
| Mro | 5.8586 |

Table S3: qRT-PCR of genes in freshly isolated cortical and spinal cord tissue. Results represent the delta delta critical threshold of cortical tissue compared to spinal cord tissue. The first seven genes were identified by microarray as enriched in cortical derived neurospheres. The bottom eight genes were identified by microarray as enriched in spinal cord derived neurospheres. Relative expression > 1 indicates greater expression in spinal cord tissue.
