# Supplementary material for: Cell Lineage and Regional Identity of Cultured Spinal Cord Neural Stem Cells and Comparison to Brain-Derived Neural Stem Cells
Source: PLoS One. 2009 Jan 16;4(1):e4213. doi: 10.1371/journal.pone.0004213 (PMC2615219; doi:10.1371/journal.pone.0004213)
Supplement: Table S4 — List of genes enriched in neuropshere derived from LeX+ cells (LeX+/LeX−>2) and genes enriched in neurospheres derived from LeX− cells (LeX+/LeX−<0.5). (0.14 MB DOC) [file pone.0004213.s004.doc]

| Genes Enriched in LeX+ derived Neurospheres |  |  |  |  |
| --- | --- | --- | --- | --- |
| **Gene Name** | **Accession Number** | **Description** | **LeX+/ LeX-** | **P- VALUE** |
| C79127 | NM_177691 | expressed sequence C79127 | 5.4 | 0 |
| Nupr1 | NM_019738 | nuclear protein 1 (Nupr1) | 5 | 0 |
| Nfatc2 | AK081853 | RIKEN full-length enriched library, clone:product:nuclear factor of activated T-cells, cytoplasmic 2, full insert sequence. | 4 | 0.02 |
| 9230117N10Rik | NM_133775 | RIKEN cDNA 9230117N10 gene | 3.8 | 0 |
| Cxcl1 | NM_008176 | chemokine (C-X-C motif) ligand 1 (Cxcl1) | 3.6 | 0.01 |
| Avil | NM_009635 | advillin (Avil) | 3.6 | 0.02 |
| Pla2g7 | NM_013737 | phospholipase A2, group VII (platelet-activating factor acetylhydrolase, plasma) (Pla2g7) | 3.5 | 0 |
| Aqp4 | NM_009700 | aquaporin 4 (Aqp4) | 3.3 | 0 |
| Olfml2a | AK037205 | RIKEN full-length enriched library, product:unclassifiable | 3.2 | 0.02 |
| Adra2a | NM_007417 | adrenergic receptor, alpha 2a (Adra2a) | 3.2 | 0 |
| Aldh1l1 | NM_027406 | aldehyde dehydrogenase 1 family, member L1 (Aldh1l1) | 3.1 | 0 |
| Fcgrt | NM_010189 | Fc receptor, IgG, alpha chain transporter (Fcgrt) | 3 | 0 |
| Agt | NM_007428 | angiotensinogen (serpin peptidase inhibitor, clade A, member 8) (Agt) | 2.9 | 0 |
| Mmd2 | NM_175217 | monocyte to macrophage differentiation-associated 2 (Mmd2) | 2.9 | 0.01 |
| Klf2 | NM_008452 | Kruppel-like factor 2 (lung) (Klf2) | 2.8 | 0.01 |
| Syn3 | AK082445 | RIKEN full-length enriched library, product:synapsin 3, | 2.8 | 0.01 |
| Ndg2 | NM_175329 | Nur77 downstream gene 2 (Ndg2) | 2.7 | 0 |
| E430002G05Rik | NM_173749 | RIKEN cDNA E430002G05 gene (E430002G05Rik) | 2.7 | 0.01 |
| Trib3 | NM_144554 | tribbles homolog 3 (Drosophila) (Trib3), transcript variant 1 | 2.7 | 0 |
| 2310050B05Rik | AK009897 | RIKEN full-length enriched library, product:unclassifiable | 2.6 | 0 |
| Cldn10 | NM_021386 | claudin 10 (Cldn10), transcript variant 2 | 2.6 | 0 |
| Klf4 | NM_010637 | Kruppel-like factor 4 (gut) (Klf4) | 2.5 | 0 |
| Odz4 | NM_011858 | odd Oz/ten-m homolog 4 (Drosophila) (Odz4) | 2.5 | 0 |
| Syne1 | NM_153399 | synaptic nuclear envelope 1 (Syne1), transcript variant 1 | 2.5 | 0.02 |
| Apoe | NM_009696 | apolipoprotein E (Apoe) | 2.5 | 0 |
| Odz4 | AK147579 | RIKEN full-length enriched library, product:odd Oz/ten-m homolog 4 (Drosophila) | 2.4 | 0 |
| Fos | NM_010234 | FBJ osteosarcoma oncogene (Fos) | 2.4 | 0.01 |
| Scg5 | NM_009162 | secretogranin V (Scg5) | 2.4 | 0 |
| Col3a1 | NM_009930 | procollagen, type III, alpha 1 (Col3a1) | 2.4 | 0.01 |
| Ndrg2 | NM_013864 | N-myc downstream regulated gene 2 (Ndrg2) | 2.4 | 0.01 |
| Crlf1 | NM_018827 | cytokine receptor-like factor 1 (Crlf1) | 2.4 | 0 |
| Slc2a5 | NM_019741 | solute carrier family 2 (facilitated glucose transporter), member 5 (Slc2a5) | 2.4 | 0.01 |
| Wnt4 | NM_009523 | wingless-related MMTV integration site 4 (Wnt4) | 2.4 | 0 |
| Plxdc1 | NM_028199 | plexin domain containing 1 (Plxdc1) | 2.3 | 0.01 |
| Susd4 | NM_144796 | sushi domain containing 4 (Susd4) | 2.3 | 0 |
| Atp1a2 | NM_178405 | ATPase, Na+/K+ transporting, alpha 2 polypeptide (Atp1a2) | 2.3 | 0.01 |
| AU041783 | NM_146102 | expressed sequence AU041783 | 2.3 | 0 |
| B830045N13Rik | NM_153539 | RIKEN cDNA B830045N13 gene (B830045N13Rik) | 2.3 | 0 |
| Olfml2a | AK030069 | RIKEN full-length enriched library, product:weakly similar to HYPOTHETICAL 48.4 KDA PROTEIN (FRAGMENT) | 2.3 | 0.03 |
| Cox8b | NM_007751 | cytochrome c oxidase, subunit VIIIb (Cox8b) | 2.3 | 0 |
| Cml1 | NM_023160 | camello-like 1 (Cml1) | 2.3 | 0 |
| Pdlim3 | NM_016798 | PDZ and LIM domain 3 (Pdlim3) | 2.3 | 0 |
| Fn3k | NM_022014 | fructosamine 3 kinase (Fn3k), transcript variant 1 | 2.3 | 0.01 |
| Gucy1a3 | NM_021896 | guanylate cyclase 1, soluble, alpha 3 (Gucy1a3) | 2.2 | 0 |
| Chac1 | NM_026929 | ChaC, cation transport regulator-like 1 (E. coli) (Chac1) | 2.2 | 0 |
| 1700125D06Rik | BU936742 | AGENCOURT_10523833 NIH_MGC_169 cDNA clone IMAGE:6704546 | 2.2 | 0.04 |
| Slc1a2 | NM_011393 | solute carrier family 1 (glial high affinity glutamate transporter), member 2 (Slc1a2) | 2.2 | 0 |
| Junb | NM_008416 | Jun-B oncogene (Junb) | 2.2 | 0.01 |
| Tspan18 | NM_183180 | tetraspanin 18 (Tspan18) | 2.2 | 0.01 |
| Hrc | NM_010473 | histidine rich calcium binding protein (Hrc) | 2.2 | 0.01 |
| Fosb | NM_008036 | FBJ osteosarcoma oncogene B (Fosb) | 2.2 | 0.02 |
| Scn3a | XM_001001601 | sodium channel, voltage-gated, type III, alpha, transcript variant 3 (Scn3a) | 2.2 | 0.04 |
| Kcnn2 | NM_080465 | potassium intermediate/small conductance calcium-activated channel, subfamily N, member 2 (Kcnn2) | 2.2 | 0.01 |
| AI646023 | NM_198860 | expressed sequence AI646023 (AI646023) | 2.1 | 0.01 |
| Rtn4r | NM_022982 | reticulon 4 receptor (Rtn4r) | 2.1 | 0 |
| Nfkbiz | NM_030612 | nuclear factor of kappa light polypeptide gene enhancer in B-cells inhibitor, zeta (Nfkbiz) | 2.1 | 0.01 |
| Hoxa11 | NM_010450 | homeo box A11 (Hoxa11) | 2.1 | 0 |
| Bmp3 | NM_173404 | bone morphogenetic protein 3 (Bmp3) | 2.1 | 0.02 |
| Phyh | NM_010726 | phytanoyl-CoA hydroxylase (Phyh) | 2.1 | 0 |
| AK053887 | AK053887 | RIKEN full-length enriched library, product:unclassifiable | 2.1 | 0.01 |
| Ctsh | NM_007801 | cathepsin H (Ctsh) | 2.1 | 0 |
| 1110046J04Rik | AK004186 | RIKEN full-length enriched library, product:unclassifiable, | 2.1 | 0.02 |
| Abca1 | NM_013454 | ATP-binding cassette, sub-family A (ABC1), member 1 (Abca1) | 2.1 | 0 |
| M6prbp1 | NM_025836 | mannose-6-phosphate receptor binding protein 1 (M6prbp1) | 2.1 | 0 |
| Lnx1 | NM_010727 | ligand of numb-protein X 1 (Lnx1) | 2.1 | 0 |
| D630004D15Rik | AK052608 | RIKEN full-length enriched library, product:unclassifiable | 2 | 0.04 |
| Igsf4d | NM_178721 | immunoglobulin superfamily, member 4 (Igsf4d) | 2 | 0.01 |
| Pfkp | BC050264 | clone IMAGE:4503748 | 2 | 0 |
| 2900019G14Rik | BC078451 | RIKEN cDNA 2900019G14 gene | 2 | 0 |
| Sned1 | NM_172463 | sushi, nidogen and EGF-like domains 1 (Sned1) | 2 | 0.01 |
| 1200009I06Rik | NM_028807 | RIKEN cDNA 1200009I06 gene (1200009I06Rik) | 2 | 0 |
| Tiam2 | NM_011878 | T-cell lymphoma invasion and metastasis 2 (Tiam2) | 2 | 0.01 |
| Socs3 | NM_007707 | suppressor of cytokine signaling 3 (Socs3) | 2 | 0 |
|  |  |  |  |  |
| Genes Enriched in LeX- derived Neurospheres |  |  |  |  |
| **GeneName** | **Accession Number** | **Description** | **LeX+/ LeX-** | **P VALUE** |
| Pcsk5 | AK086210 | RIKEN full-length enriched library, product: proprotein convertase subtilisin/kexin type 5 | 0.4 | 0 |
| AK038954 | AK038954 | RIKEN full-length enriched library, product: hypothetical protein | 0.4 | 0 |
| Spp1 | NM_009263 | secreted phosphoprotein 1 (Spp1) | 0.4 | 0 |
| B230112C05Rik | AK050937 | RIKEN full-length enriched library, product:unclassifiable | 0.4 | 0 |
| Gbp4 | NM_018734 | guanylate nucleotide binding protein 4 (Gbp4) | 0.4 | 0 |
| Dhrs2 | NM_027790 | dehydrogenase/reductase member 2 (Dhrs2) | 0.5 | 0 |
| Apln | NM_013912 | apelin (Apln) | 0.5 | 0 |
| Atf7ip2 | BC018510 | activating transcription factor 7 interacting protein 2 | 0.5 | 0 |
| Ccbe1 | BC103803 | collagen and calcium binding EGF domains 1 | 0.5 | 0 |
| Cdk6 | AK030810 | RIKEN full-length enriched library, clone: product: unclassifiable | 0.5 | 0 |
| Cpne8 | NM_025815 | copine VIII (Cpne8), transcript variant 1 | 0.5 | 0 |
| Akap12 | NM_031185 | A kinase (PRKA) anchor protein (gravin) 12 (Akap12) | 0.5 | 0 |
| Tmem20 | NM_175507 | transmembrane protein 20 (Tmem20) | 0.5 | 0 |
| Dtl | AK012919 | RIKEN full-length enriched library, clone:product: WEAKLY SIMILAR TO LETHAL(2)DENTICLELESS PROTEIN | 0.5 | 0.01 |
| Myh7 | NM_080728 | myosin, heavy polypeptide 7, cardiac muscle, beta (Myh7) | 0.5 | 0 |

Table S4: List of genes enriched in neuropshere derived from LeX+ cells (LeX+/LeX->2) and genes enriched in neurospheres derived from LeX- cells (LeX+/LeX- <0.5).
