# Supplementary material for: Cell Lineage and Regional Identity of Cultured Spinal Cord Neural Stem Cells and Comparison to Brain-Derived Neural Stem Cells
Source: PLoS One. 2009 Jan 16;4(1):e4213. doi: 10.1371/journal.pone.0004213 (PMC2615219; doi:10.1371/journal.pone.0004213)
Supplement: Table S5 — Overlap of genes in regionally specific neurospheres and ependymomas. (SC: spinal cord; ST: supratentoral). Ependymoma enriched genes identified by Taylor MD, Poppleton H, Fuller C, Su X, Liu Y, Jensen P, Magdaleno S, Dalton J, Calabrese C, Board J, Macdonald T, Rutka J, Guha A, Gajjar A, Curran T, Gilbertson RJ (2005) Radial glia cells are candidate stem cells of ependymoma. Cancer Cell 8:323–335. (0.04 MB DOC) [file pone.0004213.s005.doc]

| Genes in SC ependymoma and up in SC NS |  | Genes in ST ependymoma and up in Cortical NS |  |
| --- | --- | --- | --- |
| SC ependymoma | SC NS | ST ependymoma | Brain NS |
| CAST | Cast | AMPH | Amph |
| FLNB | Flnb | COL18A1 | Col18a1 |
| HOXA7 | Hoxa7 | EMX2 | Emx2 |
| HOXA9 | Hoxa9 | INSIG1 | Insig1 |
| HOXB6 | Hoxb6 | LHX2 | Lhx2 |
| HOXB7 | Hoxb7 | LYN | Lyn |
| HOXC10 | Hoxc10 | NR2E1 | Nr2e1 |
| HOXC6 | Hoxc6 |  |  |
| RGNEF | Rgnef |  |  |

Table S5: Overlap of genes in regionally specific neurospheres and ependymomas. (SC: spinal cord; ST: supratentoral). Ependymoma enriched genes identified by Taylor MD, Poppleton H, Fuller C, Su X, Liu Y, Jensen P, Magdaleno S, Dalton J, Calabrese C, Board J, Macdonald T, Rutka J, Guha A, Gajjar A, Curran T, Gilbertson RJ (2005) Radial glia cells are candidate stem cells of ependymoma. Cancer Cell 8:323-335.
