# Supplementary figures and images for: Cell Lineage and Regional Identity of Cultured Spinal Cord Neural Stem Cells and Comparison to Brain-Derived Neural Stem Cells
Source: PLoS One. 2009 Jan 16;4(1):e4213. doi: 10.1371/journal.pone.0004213 (PMC2615219; doi:10.1371/journal.pone.0004213)

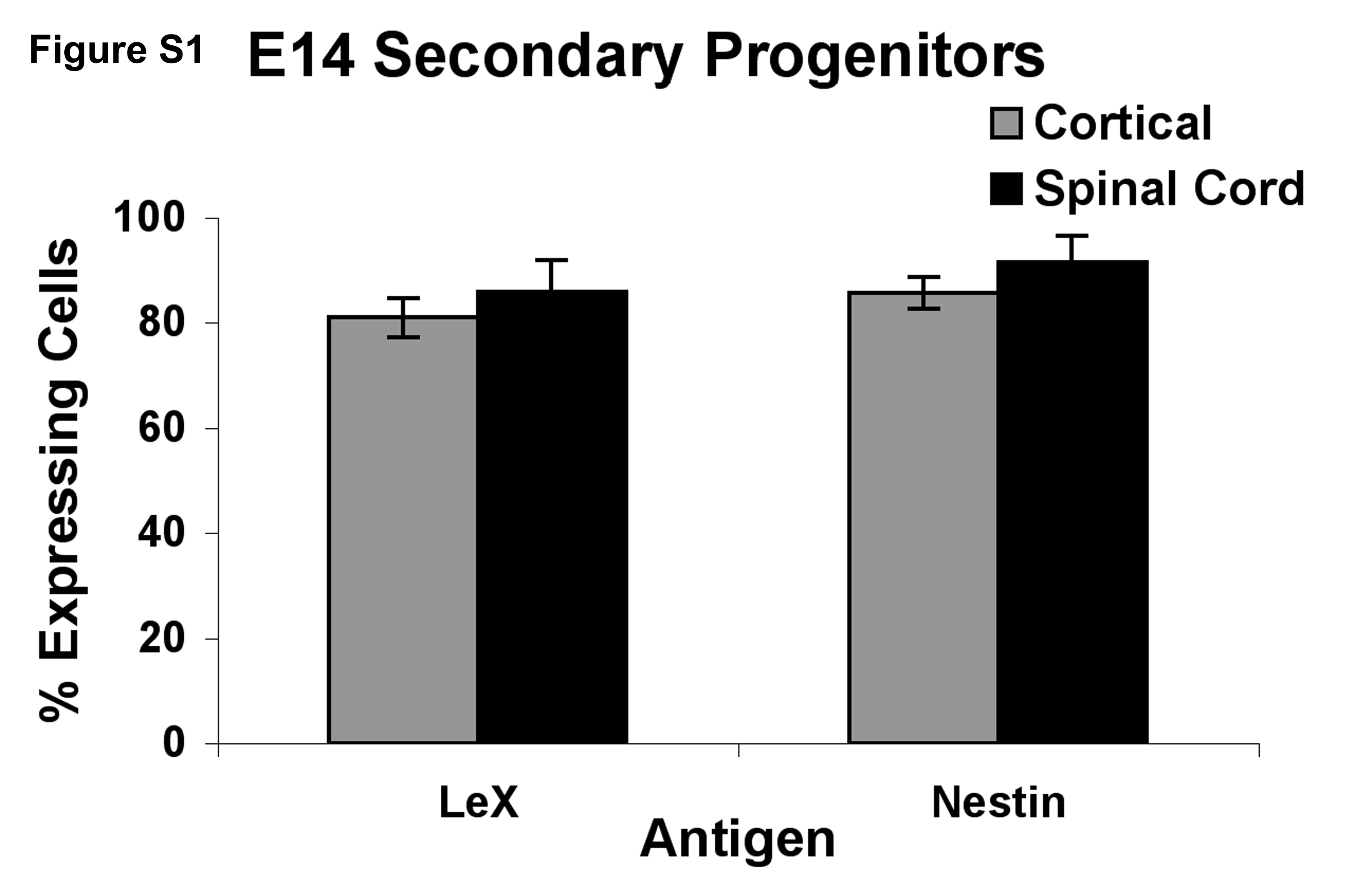

Supplement: Figure S1 — The majority of cells within cortical and spinal cord derived neurospheres express LeX and/or Nestin. E14 secondary neurospheres were dissociated and stained with antibodies to LeX or Nestin. Bars are mean+SEM. (0.44 MB TIF) [file pone.0004213.s006.tif]

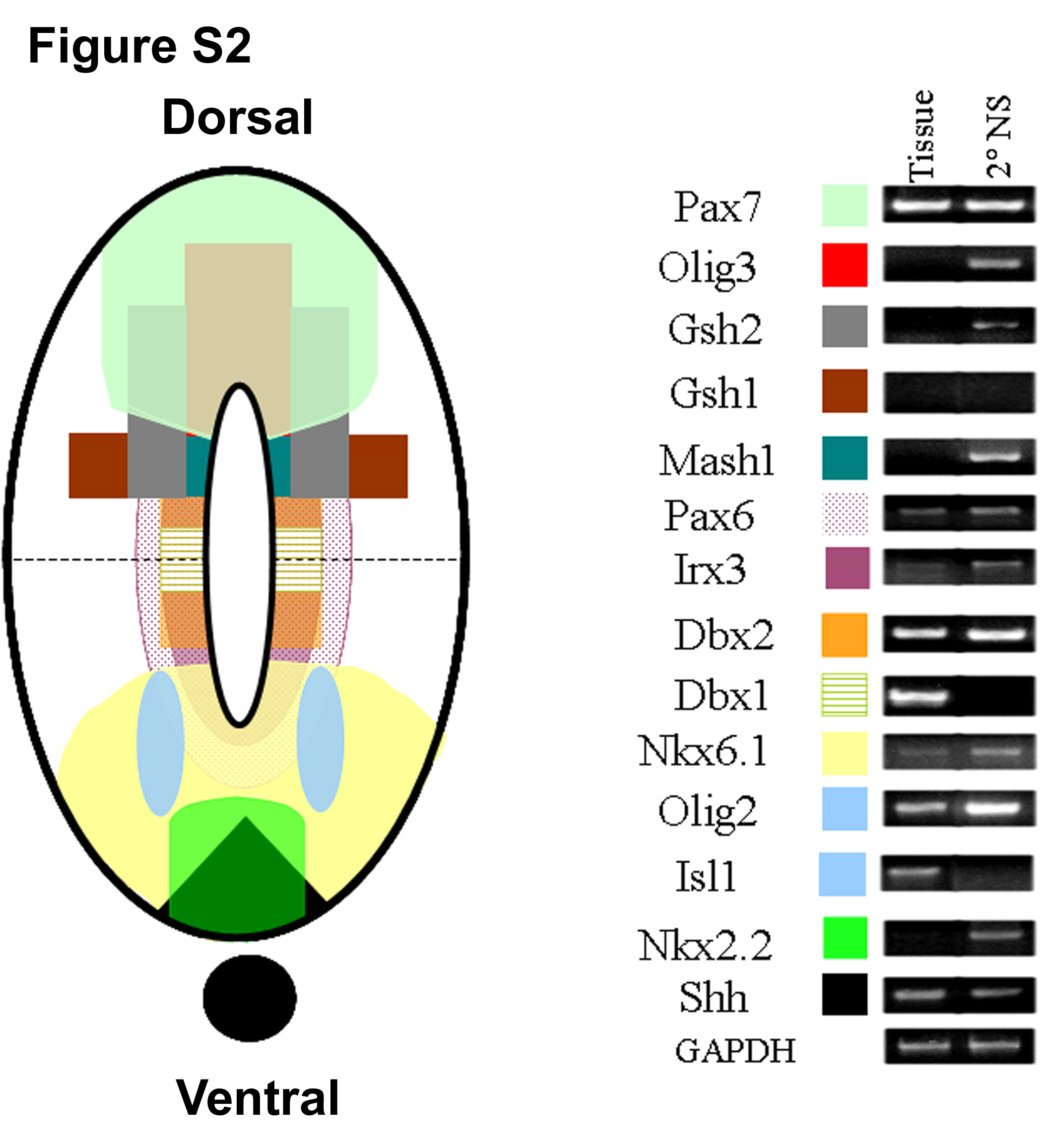

Supplement: Figure S2 — Expression of genes indicative of multiple dorsoventral regions is maintained in vitro. Gene expression was examined in primary tissue and secondary spinal cord derived neurospheres cultured at high density. Schematic represents composite of gene expression patterns throughout embryonic development, not any particular embryonic age. Genes were assessed between 25–35 cycles. Gapdh was assessed at 18 cycles. (1.55 MB TIF) [file pone.0004213.s007.tif]

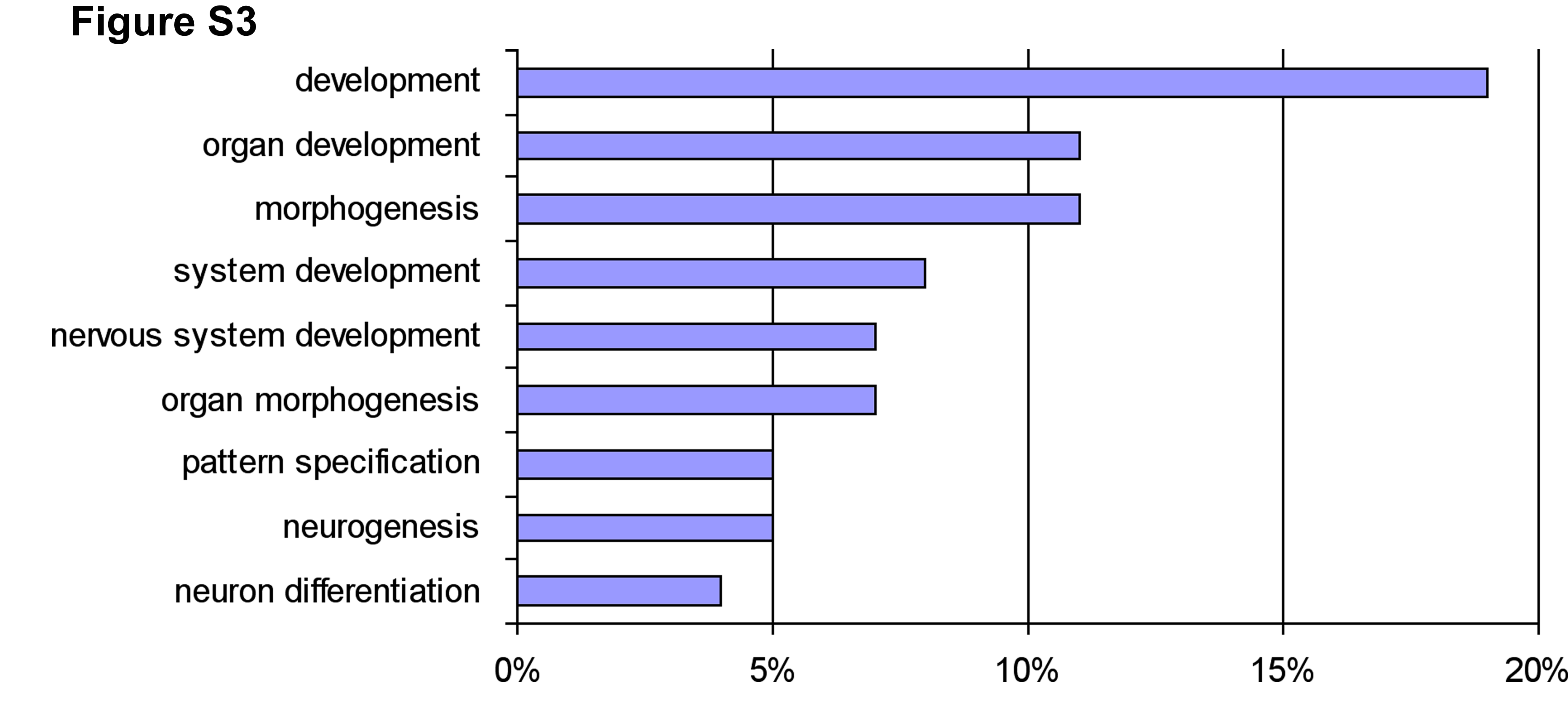

Supplement: Figure S3 — DAVID gene ontology analysis reveals global changes in biological processes between cortical and spinal cord derived neural progenitors. Most significant changes are observed generally in the development category and more specifically in pattern specification, nervous system development and neurogenesis. X axis is the percentage of genes that fit within a given functional category. (0.80 MB TIF) [file pone.0004213.s008.tif]
