# Supplementary material for: Cell Lineage and Regional Identity of Cultured Spinal Cord Neural Stem Cells and Comparison to Brain-Derived Neural Stem Cells
Source: PLoS One. 2009 Jan 16;4(1):e4213. doi: 10.1371/journal.pone.0004213 (PMC2615219; doi:10.1371/journal.pone.0004213)
Supplement: Supplementary file 6 [file pone.0004213.s001.doc]

Primer	Forward	Reverse
Abcg2	CCATAGCCACAGGCCAAAGT	GGGCCACATGATTCTTCCAC
Anxa2	AGCTCTATGATGCCGGGGTGAAG	CAGGCAAGGGCAGCTAAGATGG
Arx	GGTGAGCATCAGTCGTAGCA	AGGGTAGTGCGTCTTCTGGA
Bmi-1	AGAAGAGATTTTTATGCAGCTCA	CAACTTCTCCTCGGTCTTCA
Cav2	ATCTGCAGCCATGCTCTCTT	ACAGGATACCCGCAATGAAG
Ccng1	ATGATAGAAGTACTGACAACTGACTCT	CTAACAAATGGTCTCAGGAATCG
Egf-R	CTTTCACGCGCACTCCT	TCACGGCCTTGCAGTCT
Emx2	ATGTTTCAGCCGGCGC	TTAATCGTCTGAGGTCACATCTATTT
Fut9	TTCGCCCATTTCTAATCGTC	CTTGCTTGGCACTTCAAACA
Gapdh	CTACCCCCAATGTGTCCGTCGTG	GGGGGCCGAGTTGGGATAGG
Hoxc10	GAGCGCTATAACCGTAACGC	CTGAGGCGATTCCAGATGTT
Hoxd9	CAGCAACTTGACCCAAACAA	GGCACTTCTCCTTGCTCATC
Hoxd10	GAAGAGGTGCCCTTACACCA	TCGATTCTCTCGGCTCATCT
Irx3	CCTCTGGGTCCCTATCCAAT	TCGTCCGAGTCGCTAGTTTT
KitL	ATGAAGAAGACACAAACTTGGATTATC	TTACACCTCTTGAAATTCTCTCTCTTT
Lhx2	TCAACTGCTTCACATGCACA	TTTCCTGCCGTAAAAGGTTG
Maestro	ATGACTGTTCGAGCCTGTGT	AGCAAGCTCCAAGCCAGTTA
Melk	CTTGGATCAGAGGCAGATGTTTGGAG	GCTGTAATCTTGCATGACCCAGG
Musashi	GAGACTGACGCGCCCCAGCC	CGCCTGGTCCATGAAAGTGACG
Nestin	GGAGCGCGTGGCACACATGG	GTAGACCCTGCTTCTCCTGCTCCAG
Nr2e1	ATGCCCCGTAGACAAGACAC	GGCCCATTGTGCTATTCCTA
Ntrk2	CTGGCTGCTCCTAACCTCAC	CTTGGCCATCAGGGTGTAGT
Ncs	CAAATGTGGGGAAAAGCAGTGTCA	GCAGGGGGATGGCAATAGTAACC
Pcsk6	ATCACCACCAGGAGACGAAC	TCGACACACTTTGTGGGGTA
Sox2	GGGCTCTGTGGTCAAGTCC	CTGCGAAGCGCCTAACGTA

Table S1: List of PCR Primers
